# Development of an Analytical Method for Determination of Related Substances and Degradation Products of Cabotegravir Using Analytical Quality by Design Principles

**DOI:** 10.1021/acsomega.1c07260

**Published:** 2022-03-04

**Authors:** Lidija Kovač, Zdenko Časar, Tina Trdan Lušin, Robert Roškar

**Affiliations:** † Analytics Department, Lek Pharmaceuticals d.d., Sandoz Development Center Slovenia, Ljubljana, SI- 1526, Slovenia; ‡ Faculty of Pharmacy, University of Ljubljana, Ljubljana, SI-1000, Slovenia

## Abstract

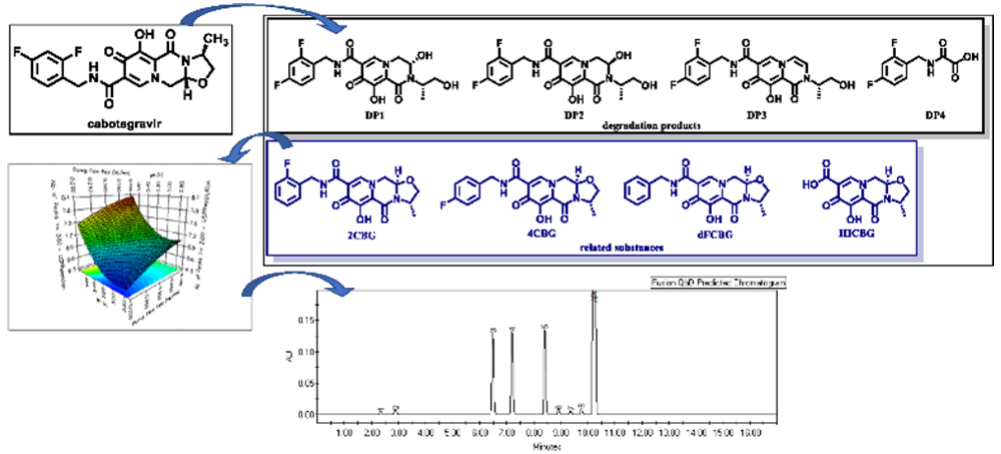

Cabotegravir is one of the newly approved human immunodeficiency virus (HIV) integrase enzyme inhibitors used for the prevention and treatment of HIV infection. It is the first approved long-acting injectable antiretroviral therapy for HIV and is also very effective in combination with rilpivirine, a non-nucleoside reverse transcriptase inhibitor. Therefore, future drug development involving cabotegravir can be expected. We developed an ultrahigh performance liquid chromatography (UHPLC) method compatible with mass spectrometry for the determination of eight cabotegravir impurities. The described method is able to differentiate cabotegravir and its related substances as well as its degradation products. Analytical quality by design principles were used for method development. The method is robust within the defined method operable design region: flow rate = 0.32–0.40 mL/min; column temperature = 30–40 °C; pH of mobile phase A = 3.25–3.75, and the final percent of acetonitrile in gradient = 50.0–60.0%. Inside the method operable design region, a working optimal point was selected: pump flow rate = 0.36 mL/min; column temperature = 35 °C; pH of mobile phase A = 3.5, and final percent of acetonitrile in gradient = 55%. Method validation was performed, and the following parameters were verified: accuracy, repeatability, linearity, response factors, detection limit, and quantification limit. All method validation results were within selected criteria. The presented method could be used for the development of new pharmaceutical products based on cabotegravir.

## 1 Introduction

**Figure 1:**
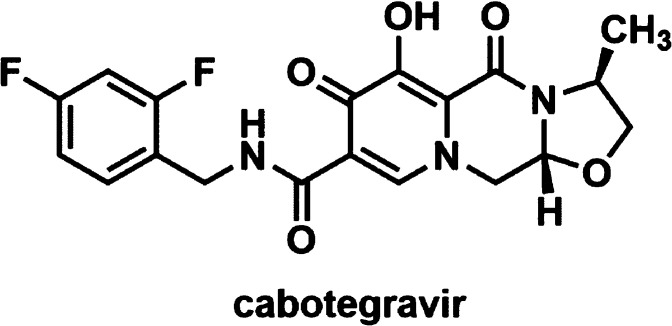
Molecular structure of cabotegravir.

Cabotegravir, chemically known as (3*S*,11*aR*)-*N*-((2,4-difluorophenyl)methyl)-6-hydroxy-3-methyl-5,7-dioxo-2,3,5,7,11,11*a*-hexahydrooxazolo(3,2-*a*)pyrido(1,2-*d*)pyrazine-8-carboxamide (Figure 1) is a second-generation integrase inhibitor, developed by Viiv Healthcare for HIV treatment and pre-exposure prophylaxis.^1^

Cabotegravir was approved by Health Canada and the European Medicines Agency in 2020 and by the U.S. Food and Drug Administration in 2021. The combination therapy with cabotegravir and rilpivirine using tablets are indicated (A) in virologically stable and suppressed adults for short-term treatment of HIV-1 infection, (B) in the assessment of cabotegravir tolerability in patients before starting of cabotegravir and rilpivirine extended-release injectable suspensions as an oral lead-in, and (C) in the case of the missed long-acting injections as oral bridging therapy. The extended-release injectable suspensions of cabotegravir and rilpivirine are used for the treatment of HIV-1 infection in virologically stable and suppressed adults to replace the current antiretroviral regimen.^2−5^ Cabotegravir has also been shown to be effective as a pre-exposure prophylaxis agent and is currently seeking approval for new therapeutical usage in United States.^6^

Cabotegravir is practically insoluble below pH 9 and slightly soluble above pH 10 in aqueous media. It has high permeability and is therefore categorized as a Biopharmaceutics Classification System (BCS) class II substance.^7^

**Figure 2:**
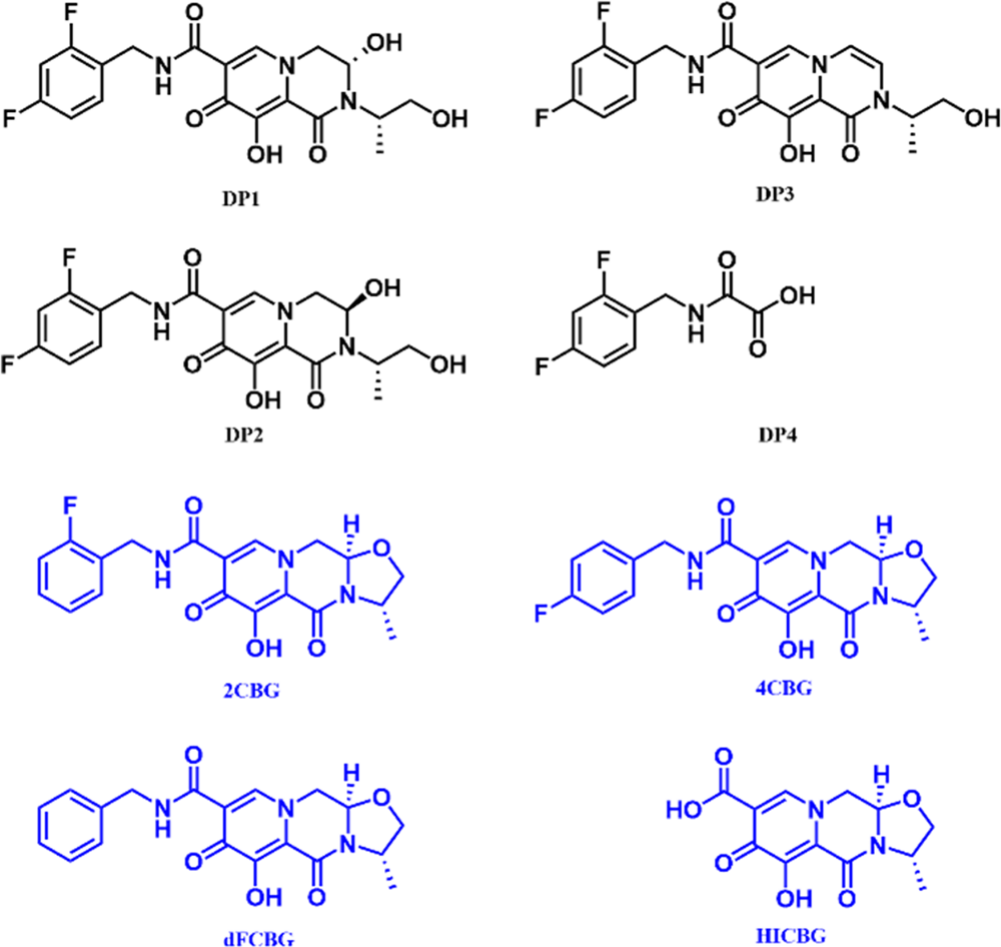
Molecular structures of known degradation products^8^ (black) and related substances (blue).

Key cabotegravir degradation products **DP1**-**DP4** (Figure 2) were recently isolated, identified, and reported by our laboratory.^8^ The purpose of our current study was the development of an analytical method for the determination of not only cabotegravir and its known degradation products **DP1**-**DP4** but also its related substances, which include desfluoro analogues **2CBG**, **4CBG**, and **dFCBG** as well as tricyclic core building block **HICBG** (Figure 2), using an analytical quality-by-design (AQbD) approach. The developed liquid chromatography method should be able to discriminate between cabotegravir and its related substances as well as its degradation products. Furthermore, the stability-indicating analytical method should be able to detect drug substance quality attributes changes during storage.^9,10^

In May 2022, the adoption of a new guideline covering AQbD topics by the International Council for Harmonization (ICH)–Q14 is expected. Therefore, the AQbD process is already being implemented in the pharmaceutical industry^11−15^ and is actively pursued in our laboratories.^16−18^ An analytical method was developed using the AQbD approach, which is an expansion of the quality-by-design (QbD) approach. It represents the development of analytical procedures using a systematic approach. The steps of the AQbD process are (i) definition of the analytical target profile (ATP), (ii) selection of critical method attributes (CMAs), (iii) risk assessment, (iv) identification of critical method parameters (CMPs), (v) screening and optimization using design of experiments (DoE), (vi) robustness testing, (vii) the definition of a method operable design region (MODR), and (viii) an establishment of the method control strategy. To the best of our knowledge, a suitable analytical method for the determination of related substances and degradation products of cabotegravir has not been reported yet. Thus, this study aims to develop an analytical method for the determination of eight cabotegravir impurities.

## 2 Results and Discussion

### 2.1 Sample Preparation

Cabotegravir is practically insoluble in aqueous solutions and poorly soluble in organic solvents such as acetonitrile (ACN) and methanol (MeOH) that are usually used in reversed-phase liquid chromatography (LC) sample preparations. Nevertheless, four common solution media for sample preparation (80% aqueous ACN, 50% aqueous ACN, 80% aqueous MeOH, and 50% aqueous MeOH) were tested. Cabotegravir was successfully dissolved up to 1 mg/mL by using 50% or 80% ACN solution as solvent. On the other hand, by using MeOH in the solvent mixture, the prepared 1 mg/mL solution of cabotegravir was turbid, and cabotegravir was only partially dissolved. Therefore, ACN was selected as an organic component for the solvent. Furthermore, as a higher portion of the organic phase in the solvent can contribute to poor peak shape due to solvent elution effect, 50% ACN solution was chosen for sample preparation. On the basis of the observed solubility of cabotegravir in the selected solvent (50% ACN), the target sample concentration of cabotegravir was set to 0.5 mg/mL.

### 2.2 Analytical Target Profile

The analytical method should be capable of separating cabotegravir and its related substances as well as its degradation products with a resolution of ≥2.0. It should be able to quantify degradation products and related substances in the range of the reporting threshold (0.05%) to 120% of the qualification threshold (0.15%) for impurities with a recovery of 70–130% and repeatability of ≤10% RSD,^19^ when the target concentration of cabotegravir is 0.5 mg/mL.

A UHPLC-UV method was chosen as the most suitable analytical technique based on the proposed ATP. The selected critical method attribute (CMA) was the resolution between peaks.

### 2.3 Method Scouting

**Figure 3:**
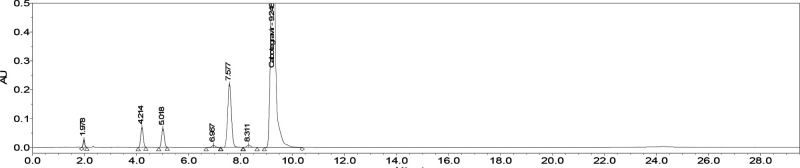
Chromatogram of cabotegravir and its related substances (**2CBG**, **4CBG**, **dFCBG**, and **HICBG**) as well as its degradation products (**DP1**, **DP2**, **DP3**, **DP4**) analyzed with initial chromatographic conditions (MP A: 0.5% formic acid:ACN (94:6, v/v); MP B: MeOH:ACN (94:6, v/v); Acquity UPLC BEH Phenyl column, 1.7 μm, 150 mm × 2.1 mm; *T*_c_ = 35 °C; flow rate = 0.3 mL/min. Gradient: *t* = 0 min, 42% B; 18 min, 42% B; 20 min, 59% B; 22 min, 89% B; 29 min, 89% B; 29,5 min, 42% B; 5.5 min equilibration).

To the best of our knowledge analytical methods for cabotegravir active pharmaceutical ingredient analysis are scare. The only recent LC-based analytical method that exists in the literature is related to the determination of cabotegravir assay in a cabotegravir and rilpivirine combination product.^20^ However, currently, there are no reported analytical methods for simultaneous determination of cabotegravir, its related substances and degradation products in the literature. An in-house method for impurities determination of a drug substance with similar physicochemical properties was the starting point for method development: mobile phase (MP) A, A = 0.5% formic acid:ACN (94:6, v/v); mobile phase B, B = MeOH:ACN (94:6, v/v); column: Acquity UPLC BEH Phenyl, 1.7 μm, 150 mm × 2.1 mm; column temperature: 35 °C; flow rate: 0.3 mL/min; autosampler temperature: 5 °C; detection wavelength: 258 nm. Gradient: *t* = 0 min, 42% B; 18 min, 42% B; 20 min, 59% B; 22 min, 89% B; 29 min, 89% B; 29.5 min, 42% B; 5.5 min equilibration. Cabotegravir and all related substances as well as all degradation products were not sufficiently separated, the cabotegravir peak also exhibited significant tailing (Figure 3).

Because of the significant tailing of cabotegravir drug substance, pH curves were predicted using the program MarvinSketch (ChemAxon, Budapest, Hungary). It was determined that cabotegravir exhibits two main species throughout the pH spectrum (Figure S1 in Supporting Information) and that it should be in un-ionized form at pH below 7; therefore, pH below 7 was used in all subsequent experiments. Some experiments of the method scouting were designed as multiple one-factor-at-a-time experiments since we decided to test two additional columns (XBridge C18, 3.5 μm, 150 mm × 4.6 mm and HSS T3, 1.8 μm, 150 mm × 2.1 mm) in combination with different pH values of mobile phase A (phosphate buffer, pH 2 and phosphate buffer, pH 5), column temperature (30 and 40 °C) and gradient time (5 and 20 min). The experiments showed that low pH around 2 in combination with the HSS T3 column, the column temperature of 30 °C, and gradient time of 20 min give better results compared to the conditions in the starting method. Results of the multiple one-factor-at-a-time experiments are presented in the Supporting Information (Figures S2–S6).

On the basis of the obtained results we decided to test mobile phases with different pH values (mobile phase A), different types of mobile phase organic modifier (mobile phase B), and different columns using a design of experiments (DoE) study. In all DoE experiments, a cubic design model and an A- and G- optimal process design were used.^21^ The focus of an A-optimal design is to minimize the average variance of predictions of all regression coefficients, and the focus of the G-optimal design is to minimize the maximum variance of all predicted values. In utilizing the DoE during the scouting phase (Table S1 and Table S2), the following variables were chosen: column used (XBridge C8, 3.5 μm, 150 mm × 4.6 mm; XBridge C18, 3.5 μm, 150 mm × 4.6 mm; BEH Phenyl, 1.7 μm, 150 mm × 2.1 mm and HSS T3, 1.8 μm, 150 mm × 2.1 mm), pH of mobile phase A (phosphate buffer, pH = 2.0; ammonium acetate buffer, pH = 4.0), organic modifier type in mobile phase B (MeOH and ACN). The described DoE was performed in two runs due to chromatographic system limitation regarding the numbers of chromatographic columns that can be used simultaneously. In the first run chromatographic columns XBridge C8 and XBridge C18 and in the second run BEH Phenyl and HSS T3 chromatographic columns were tested. The only difference between the runs was the flow rate, which was lowered for UHPLC columns BEH Phenyl and HSS T3 from 0.4 mL/min to 0.3 mL/min due to column backpressure.

**Figure 4:**
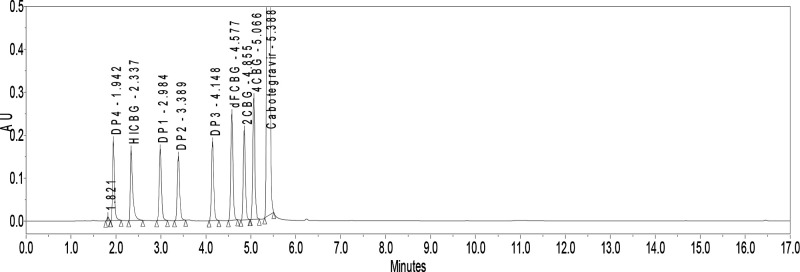
Chromatogram of the best chromatographic conditions for separation of cabotegravir, its eight impurities, and one unknown impurity from method scouting. Chromatographic conditions: MP A, phosphate buffer, pH = 2.0; MP B, ACN; Acquity UPLC BEH Phenyl column, 1.7 μm, 150 mm × 2.1 mm; *T*_c_ = 40 °C; flow rate = 0.3 mL/min. Gradient: *t* = 0 min, 30% B; 1 min, 30% B; 16 min, 90% B; 17 min, 30% B; 3 min equilibration.

During the scouting phase less strict search criteria were applied in Fusion software compared to those set in the ATP: the number of peaks with a resolution of ≥1.5 and the number of observed resolved peaks. The search for the best overall answer was carried out, and the results were evaluated based on the software calculation of cumulative desirability result (desirability is an operation of Fusion QbD software that grades all results on a desirability scale from 0 to 1, where 0 is the most undesirable result and 1 is the most desirable result). The outcome settings were two: (A) maximal number of peaks with resolution ≥ 1.5 with 8 peaks having desirability of 0 and 10 peaks having desirability of 1 and, (B) maximal number of peaks with 8 peaks having desirability of 0 and 10 peaks having desirability of 1. The most desirable result for the criteria number of observed resolved peaks was set to 10 due to the observation of an additional peak of unknown impurity in front of the **DP4** peak in some chromatograms. The most desirable results were achieved using a BEH Phenyl column, ACN (mobile phase B), and a phosphate buffer, pH = 2.0 (mobile phase A), where the method was capable of differentiating all main degradation products, related substances, and cabotegravir (Figure 4).

The cumulative desirability result was 0.6030 (target value of 1.0000 would be achieved if all 10 peaks had resolution ≥ 1.5). The overall predicted number of resolved peaks was 10, among which 9 had a resolution ≥ 1.5. An experiment was already run during the scouting DoE showing that 10 peaks were successfully resolved with 9 having a resolution ≥ 1.5 (Figure 4). Method conditions in this case were as follows: column BEH Phenyl, 1.7 μm, 150 mm × 2.1 mm; mobile phase A, phosphate buffer, pH = 2.0; mobile phase B, ACN; pump flow: 0.3 mL/min; column temperature: 40 °C;. Gradient: *t* = 0 min, 30% B; *t* = 1 min, 30% B; *t* = 16 min, 90% B, *t* = 17 min, 30% B, and followed with 3 min re-equilibration.

### 2.4 Initial Method Risk Assessment

**Figure 5:**
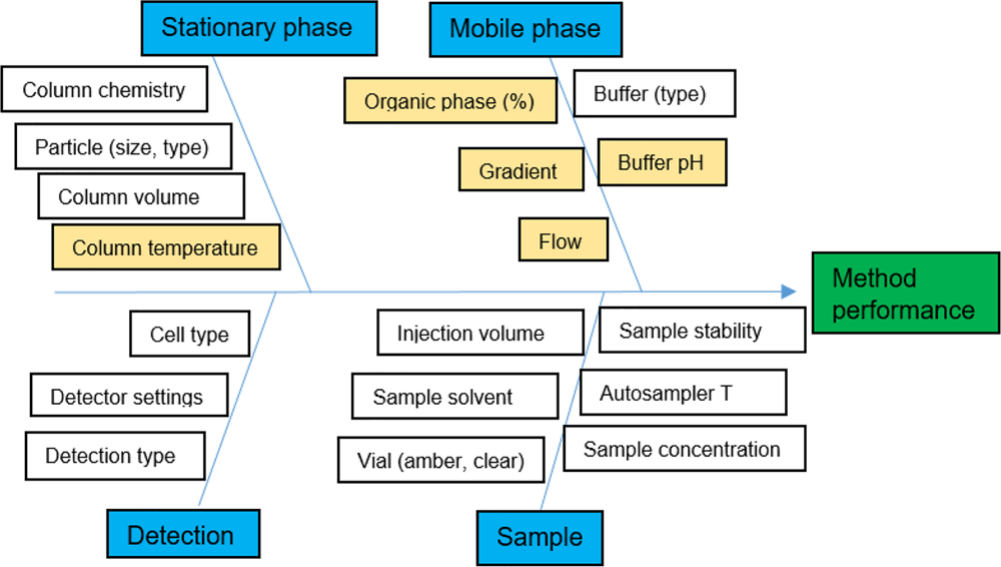
Ishikawa diagram for initial risk assessment. Factors considered as CMPs are marked with yellow color.

The “Ishikawa” diagram was used for performing the initial method risk assessment (Figure 5). On the basis of the results from method scouting we could better define critical method attributes (CMAs). The defined CMAs were (A) resolution between impurities **DP4** and **HICBG** (*R*_**DP4**,**HICBG**_ ≥ 1.5, preferably ≥2.0), (B) resolution between impurities **2CBG** and **4CBG** (*R*_**2CBG**,**4CBG**_ ≥ 1.5, preferably ≥ 2.0), (Ci) resolution between impurity **4CBG** and cabotegravir (*R*_**4CBG**,**cabotegravir**_ ≥ 1.5, preferably ≥ 2.0). Parameter groups such as stationary phase, mobile phase, detection, and sample that can affect method performance were included in the Ishikawa diagram. In each group defined in the Ishikawa diagram different respective parameters were considered. Each method parameter was assessed based on the effect on the selected CMAs (*R*_**DP4**,**HICBG**_, *R*_**2CBG**,**4CBG**_, and *R*_**4CBG**,**cabotegravir**_) which were identified in the initial experiments and the probability of their deviation from the set value. For example, the pH of mobile phase A had a significant impact on the selected resolutions which was determined during the scouting phase using DoE, and it was therefore considered as critical. On the basis of the information gained during the method scouting phase (results presented in Supporting Information), the following critical method parameters (CMPs) that affect CMAs were identified (marked in yellow background in Figure 5): column temperature, pH of mobile phase A, final percent of organic modifier, the slope of gradient, and the flow rate. The chromatographic column type as well as detection type was already selected during the scouting phase and was therefore not included in the Ishikawa diagram as CMPs. The sample preparation was also previously studied during the method development. Therefore, it was not included in the initial risk assessment after the scouting phase.

Column temperature can affect method performance, and since it was not included in our experiments during the DoE of method scouting, we believe that more information about this parameter interaction would be useful. Therefore, the column temperature was labeled as potentially critical and in need of further investigation. In the scouting phase, the combination of the following parameters: pH of mobile phase, final percent of organic modifier, gradient slope, and pump flow, considerably affected method performance. Therefore, all these parameters were included for further investigation.

### 2.5 Method Screening

**Figure 6:**
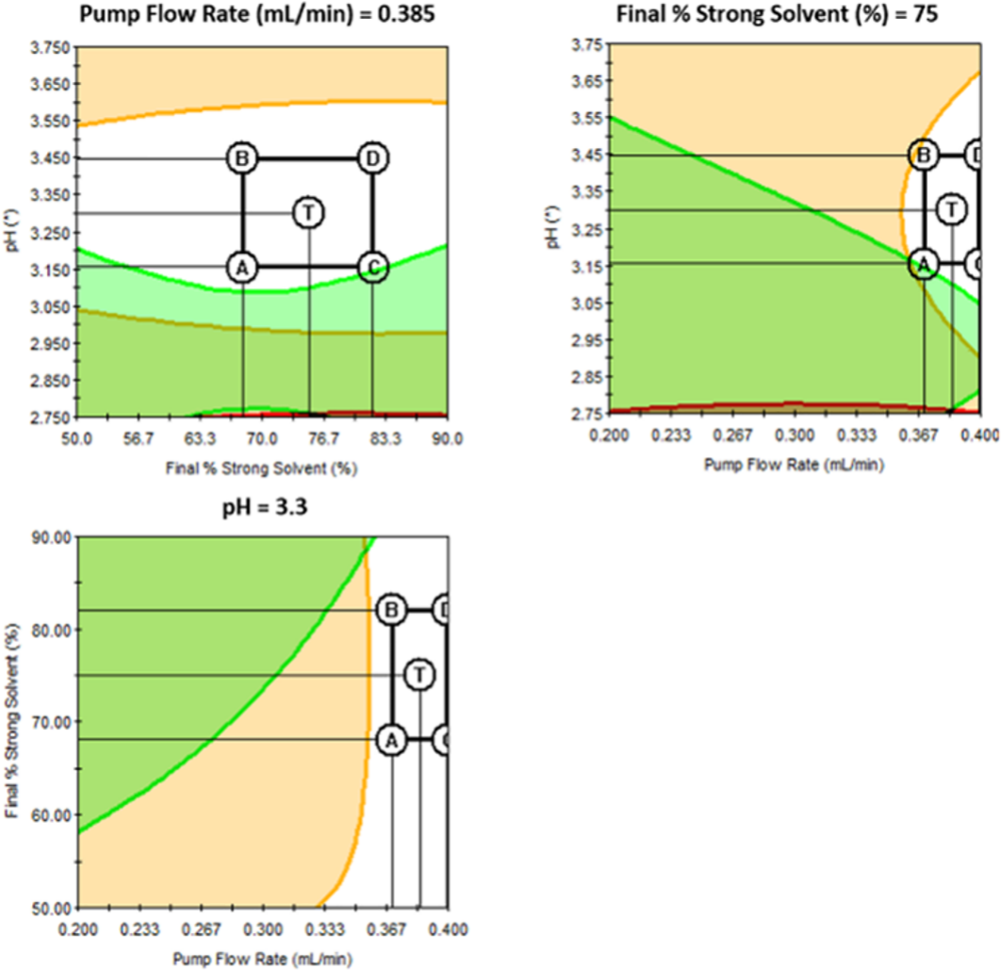
Graph representing an area where defined criteria are met in white color. Areas where the criteria are not met: green = number of peaks with resolution ≥ 2.0 less than 7; orange = number of peaks with tailing ≤ 1.2 less than 7. Acceptable range of tested conditions is marked with a black rectangle.

During the method scouting and the method screening, degradation products were not yet isolated and characterized in our laboratory.^8^ We aimed to develop an ultrahigh performance liquid chromatography method compatible with mass spectrometry (MS) that could be used also for impurity identification of cabotegravir. That is why in the method screening phase, additional MS compatible mobile phases A were tested: ammonium acetate buffer with pH values of 3.5, 4.0, and 4.5, respectively, and ammonium formate buffer with pH values of 2.75, 3.25, and 3.75, respectively. DoE studies (Table S3 and Table S4) were utilized to assess critical method parameters and their interactions, using an UPLC BEH Phenyl (1.7 μm, 150 mm × 2.1 mm) column and acetonitrile as the mobile phase B. The parameters studied in the experiments were a type of buffer and different pH values of mobile phase A (ammonium acetate buffer with pH 3.5, 4.0, 4.5, or ammonium formate buffer with pH 2.75, 3.25, and 3.75), pump flow rate (0.2, 0.3, and 0.4 mL/min, respectively), and the final percent of acetonitrile in the gradient (50–90%). The method’s constant parameters were the gradient (*t* = 0 min, 30% B; *t* = 1 min, 30% B; *t* = 16 min, 50–90% B, *t* = 17 min, 30% B and followed with 3 min re-equilibration), column temperature of 40 °C, and mobile phase B = ACN. To obtain the best result and gain better knowledge about the method, wider sets of evaluated criteria were selected and not only defined CMAs. The evaluated criteria were the number of peaks with a resolution ≥ 2.0, and the number of peaks with tailing ≤ 1.2. On the basis of the software predictions, the best method conditions (all nine separated peaks within a defined criteria), were achieved using mobile phase A as ammonium formate buffer, pH = 3.3, final percent of mobile phase B in the gradient = 75%, and flow rate = 0.385 mL/min (Figure 6).

**Figure 7:**
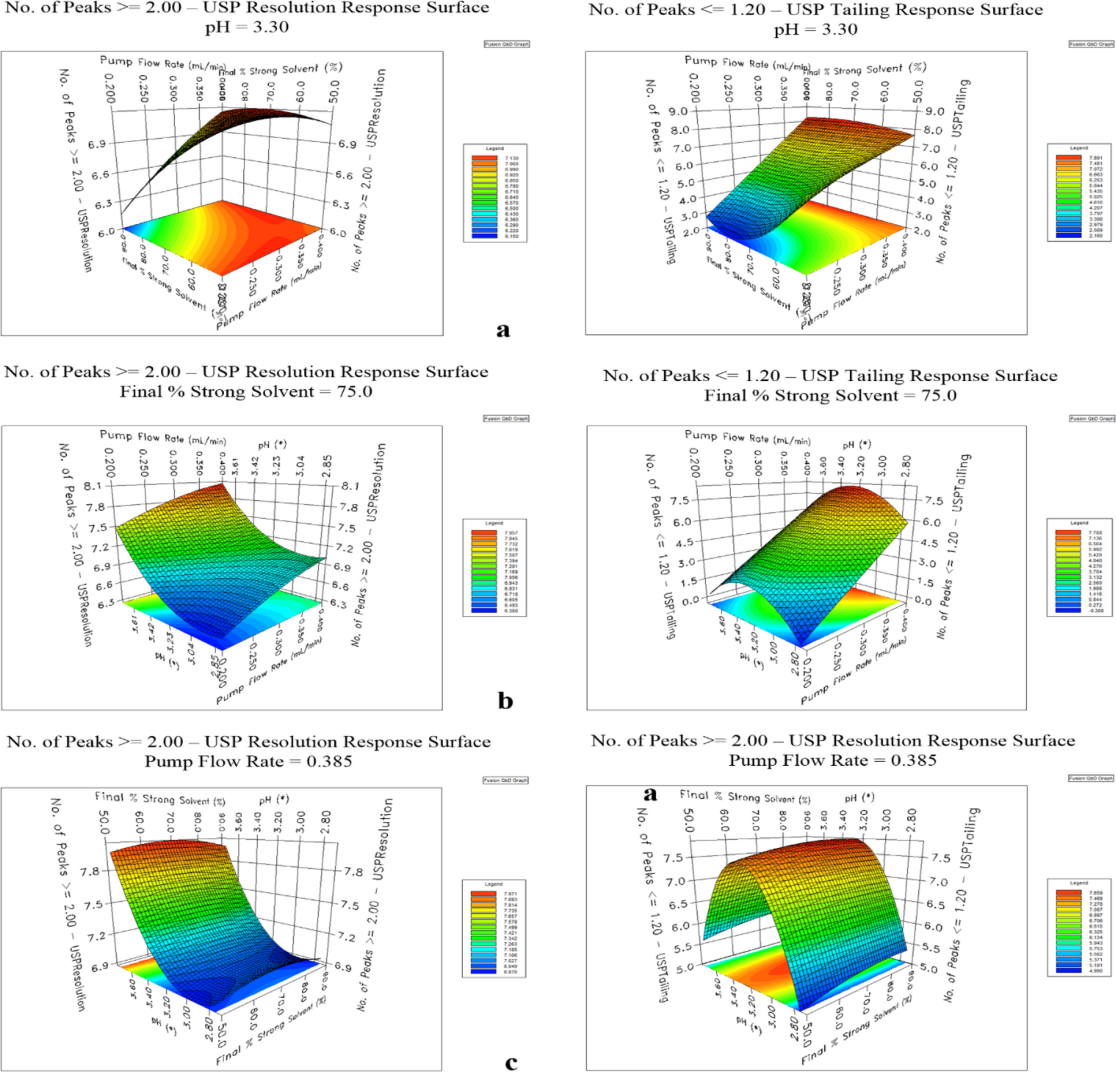
Surface plots from screening DoE representing a number of peaks with a resolution ≥ 2.0 (left) and the number of peaks with tailing ≤ 1.2 (right): (a) in relation to pump flow rate and final % of strong solvent (mobile phase B) at pH of 3.3; (b) in relation to pump flow rate and pH at final % of strong solvent (mobile phase B) of 75%, and (c) in relation to final % of strong solvent (mobile phase B) and pH at pump flow rate of 0.385 mL/min.

Method models were designed based on the results of the performed DoE study and are presented in Table 1. The statistical evaluations of model equations were performed by using analysis of variance (ANOVA). They showed good statistical significance with *F*-ratios > 4.00 and acceptable fit (*R*^2^ and LOF analysis).^21^ Established interactions of parameters and their influence on the results showed a nonlinear relation between parameters (Figure 7a–c).

**Table 1:** Method Model Equations Based on the DoE Study from Screening

observed criteria	model^a^	ANOVA^b^
no. of peaks with resolution ≥ 2.0	*y* = 6.983 + 0.230(*A*) – 0.229(*B*) + 0.516(*C*) – 0.074(*A*)^2^ – 0.085(*B*)^2^ + 0.405(*C*)^2^ + 0.266(*AB*)	*R*^2^ = 0.9981
		adj. *R*^2^ = 0.9964
		*F*-ratio = 595.0846
no. of peaks with tailing ≤ 1.2	*y* = 5.353 + 2.857(*A*) – 0.627(*B*) + 0.318(*C*) + 0.745(*B*)^2^ −2.196(*C*)^2^ + 0.920 (*AB*) – 1.015 (*A*(*B*)^2^)	*R*^2^ = 0.9681
		adj. *R*^2^ = 0.9434
		*F*-ratio = 39.0792
		MS-LOF = 0.0153 (threshold 0.3917)

a *y* = observed criteria, *A* = pump flow rate, *B* = final % of mobile phase B in gradient, *C* = pH (pH of mobile phase A).

b Regression ANOVA statistics: MS-LOF = mean square lack-of-fit, adj. = adjusted.

### 2.6 Method Optimization

All selected CMAs were already met during screening experiments. We decided to employ an additional DoE study in the method optimization phase to establish the influence of column temperature in combination with other CMPs on CMAs as the temperature effect was not investigated yet. In this phase, we decided to monitor the CMP’s effect only on the defined CMAs to ensure calculations of appropriate method model equations. The DoE study parameters (Table S5) were selected according to the initial method risk assessment (see section 2.4) and additional knowledge obtained during the method screening phase: pH of mobile phase A (2.75–3.75), column temperature (30–50 °C), final percent of mobile phase B in the gradient (50–90%), and pump flow rate (0.2–0.4 mL/min). CMAs as defined in the initial risk assessment were selected: resolution between impurities **DP4** and **HICBG** (*R*_**DP4**,**HICBG**_ ≥ 2.0), resolution between impurities **2CBG** and **4CBG** (*R*_**2CBG**,**4CBG**_ ≥ 2.0), resolution between impurity **4CBG** and cabotegravir (*R*_**4CBG**,**cabotegravir**_ ≥ 2.0). The performed DoE study enabled us to calculate method model equations for each selected parameter (CMAs). The calculated method models were statistically evaluated using ANOVA^22^ (Table 2). All models showed good statistical significance (*P*-values < 0.05, *F*-ratios > 4.00). In addition, low lack-of-fit (LOF) values and high *R*^2^ values showed good fitting models.

**Table 2:** Method Models of CMAs Based on Optimization DoE Study

model coefficients^a^		regression ANOVA statistics^b^
CMA = *R*_**DP4**,**HICBG**_		
+3.911	+0.131(*AC*)	*R*^2^ = 1.0000, adj. *R*^2^ = 0.9999, *F*-ratio = 26824.9324
+0.012(*A*)	–0.073(*AD*)	
–0.461(*C*)	–0.240(*CD*)	
+1.855(*D*)	–0.008((*A*)^2^*B*)	
–0.033(*B*)^2^	–0.048((*A*)^2^*C*)	
+0.045(*C*)^2^	+0.122((*A*)^2^*D*)	
–0.896(*D*)^2^	+0.084(*ACD*)	
CMA = *R*_**2CBG**,**4CBG**_		
+2.955	+0.042(*AC*)	*R*^2^ = 0.9995, adj. *R*^2^ = 0.9990, *F*-ratio = 1889.5476, MS-LOF = 0.0005
+0.212(*A*)	+0.051(*BC*)	
–0.492(*B*)	+0.010(*BD*)	
–0.145(*A*)^2^	–0.024(*CD*)	
+0.136(*B*)^2^	–0.039((*A*)^2^*C*)	
–0.028(*C*)^2^	–0.022((*A*)^2^*D*)	
–0.035(*D*)^2^	+0.010(*ABC*)	
+0.026(*AB*)	+0.005(*BCD*)	
CMA = *R*_**4CBG**,**cabotegravir**_		
+0.175	–0.007(*AC*)	*R*^2^ = 0.9999 adj. *R*^2^ = 0.9997, *F*-ratio = 5330.2813, MS-LOF = 0.0003
–0.055(*A*)	–0.002(*AD*)	
+0.092(*B*)	–0.001(*BC*)	
+0.009(*C*)	+0.001(*BD*)	
+0.004(*D*)	+0.005(*CD*)	
+0.033(*A*)^2^	+0.006((*A*)^2^C)	
+0.003(*B*)^2^	+0.003((*A*)^2^*D*)	
+0.006(*C*)^2^	–0.004(*ABC*)	
+0.005(*D*)^2^	–0.001(*ACD*)	
–0.034(*AB*)	+0.001(*BCD*)	

a *A* = pump flow rate, *B* = final % of mobile phase B, *C* = column oven temperature, *D* = pH (pH of mobile phase A).

b MS-LOF = mean square lack-of-fit, adj. = adjusted.

### 2.7 Robustness Study and Method Operable Design Region

**Figure 8:**
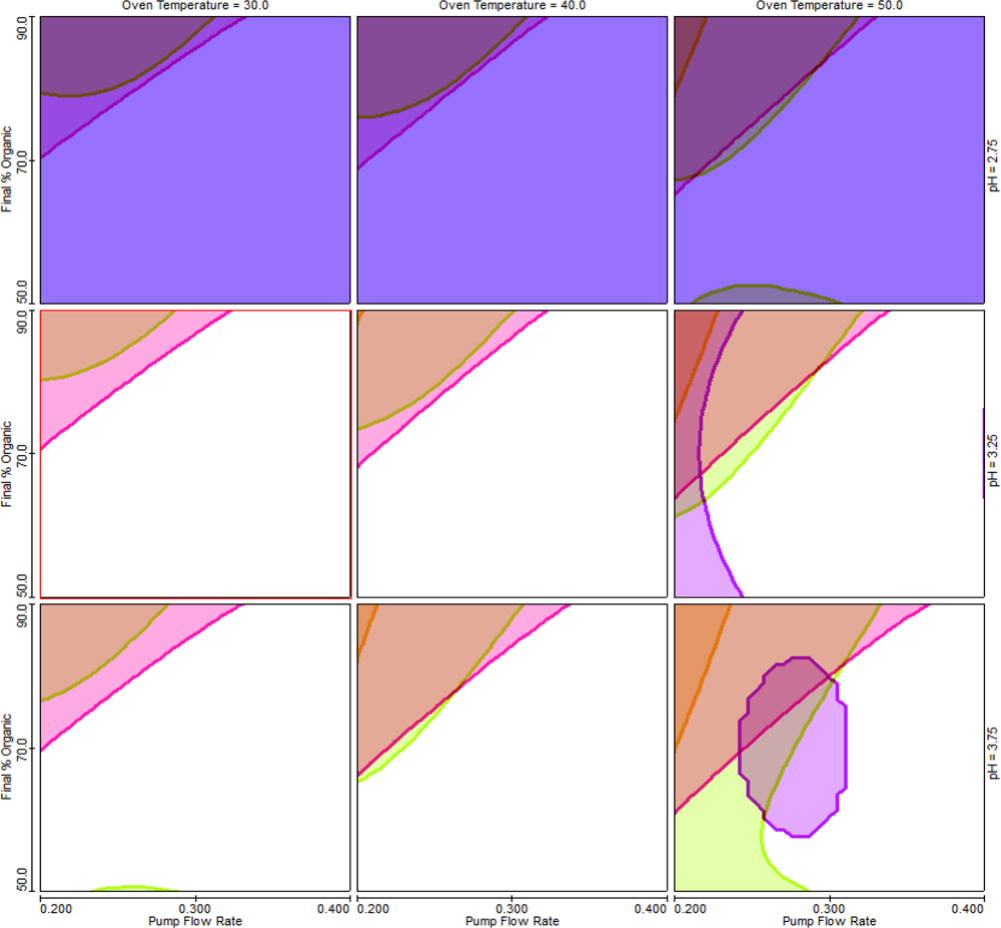
Trellis graphs from DoE study to establish CMA models and robustness testing. Graph is showing by white color an area where defined criteria are met, the design space. Other colors represent areas where the criteria are not met: dark blue = *R*_**DP4**,**HICBG**_ ≤ 2.0, purple = *C*_pk_ (*R*_**DP4**,**HICBG**_) ≤ 1.33; light blue = *R*_**2CBG**,**4CBG**_ ≤ 2.0, orange = *C*_pk_ (*R*_**2CBG**,**4CBG**_) ≤ 1.33; pink = *R*_**4CBG**,**cabotegravir**_ ≤ 2.0, lime = *C*_pk_ (*R*_**4CBG**,**cabotegravir**_) ≤ 1.33, *x* = pump flow rate (0.2–0.4 mL/min); *y* = final percent of acetonitrile in gradient (50–90%) at pH of 2.75 (top line), 3.25 (middle line), and 3.75 (bottom line); and column temperature of 30 (left column), 40 (middle column), and 50 °C (right column).

**Figure 9:**
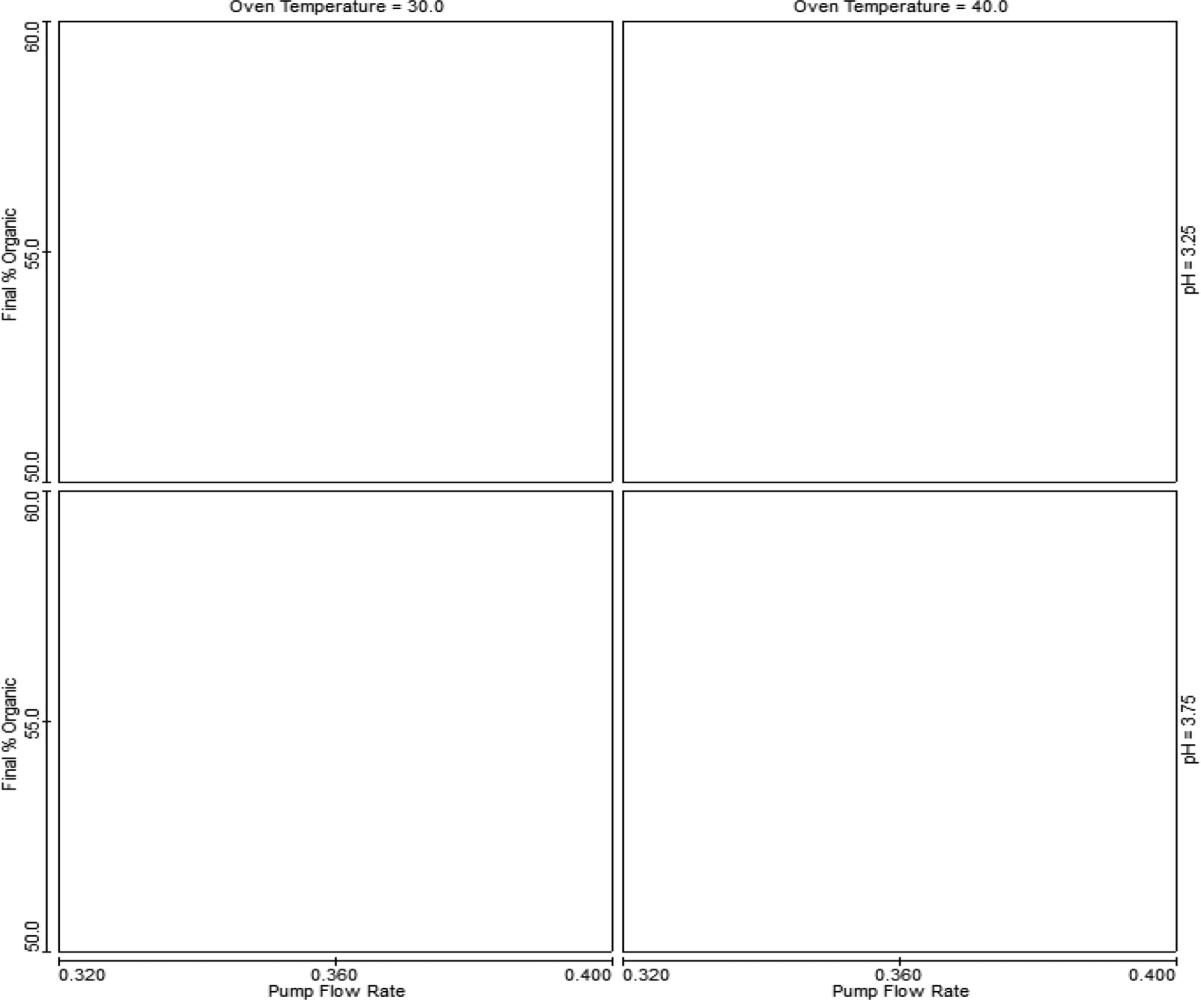
Trellis graphs of the MODR. White color represents that all defined criteria are met. *x* = pump flow rate (0.32–0.4 mL/min); *y* = final percent of acetonitrile in gradient (50–60%) at pH of 3.25 (top line) and 3.75 (bottom line); and column temperature of 30 °C (left column) and 40 °C (right column).

The robustness study for all selected CMAs using the Fusion QbD robustness simulator was done. In Fusion QbD software the system robustness is quantified using process capability indices (*C*_p_, *C*_pk_). For all selected CMAs, *C*_pk_ was used as a capability indicator with a lower specification limit set (LSL) at 1.33, meaning 99.99% of measurements will fall inside the specification limits. The calculated *C*_pk_ after running the Monte Carlo simulation is presented in graphs (Figure 8 and Figure 9).

The CMA *R*_**DP4**,**HICBG**_ ≥ 2.0 was not met, when the pH of the mobile phase A was 2.75, therefore this pH was not further considered when defining the MODR. The CMAs *R*_**2CBG**,**4CBG**_ and R_**4CBG**,**cabotegravir**_ were below 2.0 at a lower pump flow rate and higher final percent of mobile phase B in the gradient.

A MODR (control space), for which all three critical resolutions (*R*_**DP4**,**HICBG**_, *R*_**2CBG**,**4CBG**_, and *R*_**4CBG**,**cabotegravir**_) were suitable was established based on the CMA models and robustness simulations. The MODR, where the method is robust has the following parameters: flow rate = 0.32–0.40 mL/min; column temperature = 30–40 °C; pH of MF A = 3.25–3.75; final percent of mobile phase B in gradient = 50.0–60.0% (Figure 9). An experiment to confirm the predicted values for all borderline points was performed. All experimentally determined CMAs correlated well with the predicted values (Table S6–Table S26) and therefore confirmed the models.

**Figure 10:**
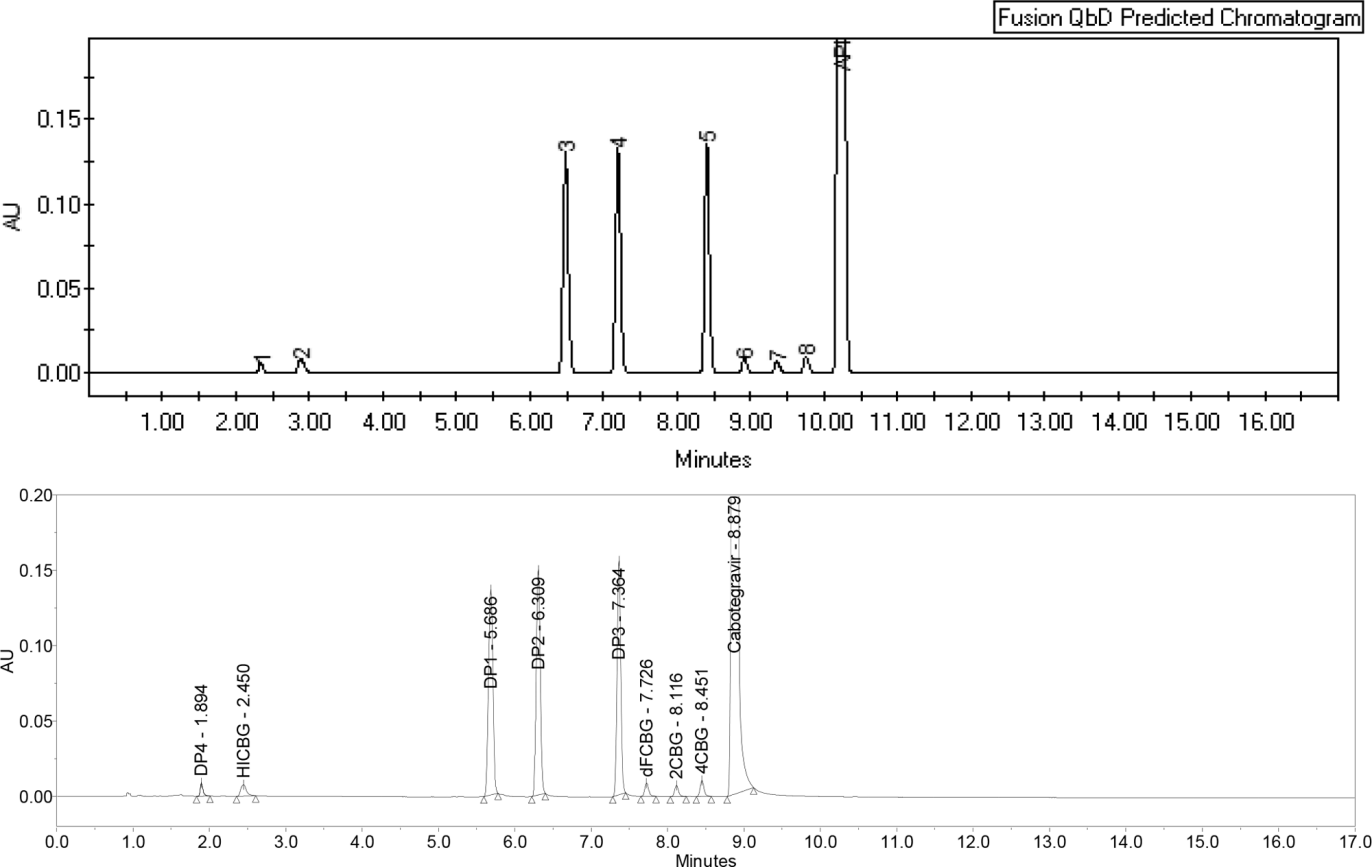
Fusion QbD software predicted chromatogram (above) and experimental chromatogram (below). Chromatographic conditions: MP A, ammonium formate buffer, pH = 3.5; MP B, ACN; UPLC BEH Phenyl column 1.7 μm, 150 mm × 2.1 mm; flow rate = 0.36 mL/min; *T*_c_ = 35 °C. Gradient: *t* = 0 min, 20% B, *t* = 1 min, 20% B, *t* = 16 min, 55% B, *t* = 17 min, 20% B, re-equilibration = 3 min.

Inside the defined MODR, a working optimal point was selected: pump flow rate = 0.36 mL/min, column temperature = 35 °C, pH of mobile phase A = 3.5, and final percent of acetonitrile in gradient = 55%. The software predicted CMAs at working optimal points are *R*_**DP4**,**HICBG**_ = 4.9, *R*_**2CBG**,**4CBG**_ = 3.5, and *R*_**4CBG**,**cabotegravir**_ = 3.2. Experimentally determined values were *R*_**DP4**,**HICBG**_ = 5.8, *R*_**2CBG**,**4CBG**_ = 3.6, and *R*_**4CBG**,**cabotegravir**_ = 4.7 (Figure 10).

### 2.8 Final Risk Assessment and Control Strategy

As demonstrated during the method optimization phase (Figure 8), critical method parameters were pH of mobile phase A, final percent of mobile phase B in the gradient, pump flow rate, and column temperature. The most critical parameters for resolution between **2CBG** and **4CBG** as well as between **4CBG** and cabotegravir are the pump flow rate and final percent of mobile phase B in the gradient, which are recommended to be maintained at the selected working optimal point. The least critical among the CMPs is the column temperature. The resolution between related substances **2CBG** and **4CBG** and between related substances **4CBG** and cabotegravir appear to be the most affected by the change of parameters out of the three CMAs. Therefore, these two resolutions can be defined as a good criterion for system suitability.

### 2.9 Analytical Method Validation

#### 2.9.1 Accuracy and Repeatability of the Method

The accuracy of the method was investigated by measuring spiked samples in the range from the quantification limit (0.05%) to 120% of the qualification threshold (0.15%). All eight impurities were added so that the added concentration of each impurity in the final solution was around 0.05%, 0.15%, and 0.18% according to cabotegravir in the sample solution (cabotegravir, ∼0.5 mg/mL). All measurements were done in three replicates. The accuracy of the method is expressed as recovery in percentage. The repeatability of the method is expressed as RSD. All results are presented in Table 3.

**Table 3:** Accuracy and Repeatability Results from Analytical Method Validation

impurity	replicate	%	added (μg/mL)	found (μg/mL)	% recovery	% RSD
**DP1**	1	0.05	0.2266	0.2094	92.42	1.13
	2	0.15	0.6798	0.6264	92.15	0.61
	3	0.18	0.8157	0.7635	93.60	1.07
**DP2**	1	0.05	0.2389	0.2384	99.81	1.34
	2	0.15	0.7166	0.7185	100.27	0.50
	3	0.18	0.8600	0.8697	101.14	0.95
**DP3**	1	0.05	0.2628	0.2909	110.71	2.47
	2	0.15	0.7883	0.8878	112.63	0.28
	3	0.18	0.9459	1.0653	112.62	0.30
**DP4**	1	0.05	0.3785	0.2966	78.37	0.88
	2	0.15	0.9378	0.9932	105.91	0.69
	3	0.18	1.1355	1.0348	91.13	1.45
**dFCBG**	1	0.05	0.2823	0.3133	110.99	1.04
	2	0.15	0.8468	0.9144	107.99	0.29
	3	0.18	1.0161	1.0948	107.75	0.87
**2CBG**	1	0.05	0.2758	0.2857	103.60	1.57
	2	0.15	0.8273	0.8824	106.67	0.56
	3	0.18	0.9927	1.2140	122.30	0.61
**4CBG**	1	0.05	0.3038	0.3392	111.67	4.31
	2	0.15	0.9113	1.0279	112.81	1.22
	3	0.18	1.0935	1.2431	113.68	1.74
**HICBG**	1	0.05	0.2598	0.2701	103.99	1.91
	2	0.15	0.7793	0.8263	106.03	0.56
	3	0.18	0.9351	0.9978	106.70	0.93

For the level of impurities below 0.2%, the set criteria for accuracy of the method was recovery of 70–130% and RSD for recovery ≤ 10%.^19^ With regards to this criteria, the method was found to be accurate and precise.

#### 2.9.2 Determination of Detection Limit

Detection limit was confirmed by six injections of cabotegravir and all eight impurities solutions with a concentration of 0.025% (*c* = 0.125 μg/mL) according to cabotegravir concentration in the sample solution (*c* = 0.5 mg/mL). All results are presented in Table 4.

**Table 4:** Detection Limit Results from Analytical Method Validation

analyte	% LOD	concn (μg/mL)	avg S/N ratio (*n* = 6)
**cabotegravir**	0.025	0.125	74.8
**DP1**	0.025	0.125	50.0
**DP2**	0.025	0.125	57.7
**DP3**	0.025	0.125	115.0
**DP4**	0.025	0.125	12.5
**dFCBG**	0.025	0.125	96.8
**2CBG**	0.025	0.125	59.0
**4CBG**	0.025	0.125	60.4
**HICBG**	0.025	0.125	46.9

LOD was confirmed for the solution with concentration giving a signal-to-noise ratio ≥ 3:1.^19^ The signal-to-noise ratios for cabotegravir and all impurities were found to be higher than the set acceptance criteria for the detection limit.

#### 2.9.3 Determination of Quantification Limit

Quantification limit (QL) was confirmed by six injections of cabotegravir and all eight impurities solutions with a concentration of 0.05% (*c* = 0.25 μg/mL) according to cabotegravir concentration in the sample solution (*c* = 0.5 mg/mL). All results are presented in Table 5.

**Table 5:** Quantification Limit Results from Analytical Method Validation

analyte	% LOQ	concn (μg/mL)	avg S/N ratio (*n* = 6)	RSD area_(*n*=6)_
**cabotegravir**	0.05	0.25	170.4	0.00%
**DP1**	0.05	0.25	104.0	0.42%
**DP2**	0.05	0.25	123.3	2.25%
**DP3**	0.05	0.25	222.7	2.75%
**DP4**	0.05	0.25	25.8	2.80%
**dFCBG**	0.05	0.25	204.5	1.41%
**2CBG**	0.05	0.25	133.8	2.29%
**4CBG**	0.05	0.25	123.0	1.72%
**HICBG**	0.05	0.25	88.9	5.61%

LOQ was confirmed for the solution with concentration giving a signal-to-noise ratio ≥ 10:1 and RSD area (*n* = 6) ≤ 10%.^19^ The signal-to-noise ratios for cabotegravir and all impurities were found to be much higher than the set acceptance criteria for the quantification limit. All RSD values were within acceptance criteria.

#### 2.9.4 Response Factors

Response factors were calculated based on the determined slope of each impurity versus the determined slope of cabotegravir. All results are presented in Table 6.

**Table 6:** Response Factor (*F*) Results from Analytical Method Validation

analyte	*F*
**DP1**	1.16
**DP2**	1.12
**DP3**	0.99
**DP4**	0.33
**dFCBG**	1.09
**2CBG**	1.01
**4CBG**	1.03
**HICBG**	0.65

#### 2.9.5 Linearity of the Method

The linearity of the method was determined using eight different solutions of impurities and cabotegravir, with concentrations ranging from 0.05% (QL) to 0.20% of the concentration of cabotegravir in the sample (*c* = 0.5 mg/mL). All measurements were done in two replicates. All summary results are presented in Table 7. More detailed linearity results are presented in the Supporting Information (Tables S27–S35).

**Table 7:** Linearity Results for Cabotegravir and All Eight Impurities from Analytical Method Validation

analyte	regression line	Pearson correlation coefficient (*r*)
**cabotegravir**	*y* = 24927*x* – 1307	0.999
**DP1**	*y* = 28984*x* – 558	1.000
**DP2**	*y* = 27865*x* – 63	1.000
**DP3**	*y* = 24753*x* – 300	1.000
**DP4**	*y* = 8303*x* – 58	1.000
**dFCBG**	*y* = 27223*x* – 487	1.000
**2CBG**	*y* = 25057*x* – 719	1.000
**4CBG**	*y* = 25708*x* – 863	1.000
**HICBG**	*y* = 16291*x* – 1090	0.998

For linearity of the method, the selected criteria was the Pearson correlation coefficient: *r* ≥ 0.998.^19^ With regard to the criteria for linearity of the method, the method was found to be linear within the defined range for all tested substances.

## 3 Conclusions

The first reversed-phase UHPLC analytical method for the determination of cabotegravir and its related substances as well as its degradation products was developed using an AQbD approach.

Critical method attributes (CMAs) were identified, and a mathematical model was established with regards to the critical method parameters (CMPs). A robust analytical method region was proposed inside the design region–control space, also known as method operable design region: flow rate = 0.32–0.40 mL/min, column temperature = 30–40 °C, pH of mobile phase A = 3.25–3.75, and final percent of mobile phase B in gradient = 50–60%. The mathematical model permits a better understanding of the influence of the method parameters on the results. The mathematical model was confirmed with the verification experiment. All predicted CMAs at the edges of the MODR correlate well with the experimental data. The developed analytical method was validated in terms of accuracy, repeatability, linearity, determination of response factors, determination of detection limit, and determination of quantification limit. The developed method achieved the analytical target profile, which was established at the beginning of the AQbD process.

The proposed UHPLC method can separate four main cabotegravir degradation products (**DP1**, **DP2**, **DP3**, and **DP4**) and four related substances (**HICBG**, **dFCBG**, **2CBG**, and **4CBG**). MS compatibility of the method enables an easy transition between different detection methods, which facilitates the identification of other potential cabotegravir degradation products.

## 4 Materials and Methods

### 4.1 Chemicals and Reagents

Cabotegravir, its related substances and cabotegravir degradation products were synthesized/isolated in Lek (Mengeš, Slovenia).^8,23^ Methanol (MeOH) and acetonitrile (ACN), both gradient grade were obtained from J.T. Baker now part of Avantor (Radnor, PA, USA). Analytical grade formic acid, glacial acetic acid, ammonium acetate, hydrochloric acid (HCl), and sodium hydroxide (NaOH) both Titrisol solutions were obtained from Merck KGaA (Darmstadt, Germany). Ammonium formate was obtained from Honeywell (Charlotte, NC, USA). Ultrapure water was obtained by a Milli-Q system from Merck Millipore (Burlington, MA, USA).

### 4.2 Equipment and Software

LC method development and analyses were performed on Acquity UPLC H-Class systems (Waters, Millford, MA, USA) equipped with a quaternary solvent manager (QSM), sample manager with a flow-through needle (SM-FTN), and either photodiode array (PDA) or tunable ultraviolet (TUV) optical detector. LC systems were equipped with Empower 3 data software (Waters, Millford, MA, USA).

Chromatography columns used during AQbD development were as listed: XBridge C8, 3.5 μm, 150 mm × 4.6 mm; XBridge C18, 3.5 μm, 150 mm × 4.6 mm; Acquity UPLC BEH Phenyl, 1.7 μm, 150 mm × 2.1 mm and Acquity UPLC HSS T3, 1.8 μm, 150 mm × 2.1 mm (Waters, Millford, MA, USA). AQbD was done with S-Matrix Fusion QbD Pro 9.8 (S-Matrix, Eureka, CA, USA). Cabotegravir drug substance and its impurities were weighed within a ventilated balance enclosure OK 15 (Iskra Pio, Šentjernej, Slovenia) on either an XP4002S precision balance, XP205 DeltaRange analytical balance, AX205 DeltaRange analytical balance, or MX5 microbalance (Mettler Toledo, Columbus, OH, USA). pH was measured using a SevenMulti pH meter (Mettler Toledo, Columbus, OH, USA). Pipettes used were Handystep electronic repetitive pipettes (Brand, Wertheim, Germany). Ultrasonic baths used were Branson 8510 (Emerson Electric, St. Louis, MO, USA), Sonic 10 and Sonic 20 (Iskra Pio, Šentjernej, Slovenia). Stress testing was done in a BF 720 standard incubator (Binder, Tuttlingen, Germany).

### 4.3 Final UHPLC Method Conditions

In the working point, the following final method conditions were selected: UPLC BEH Phenyl 1.7 μm, 150 mm × 2.1 mm column; mobile phase A = ammonium formate buffer (pH = 3.5; 10 mM), pH adjusted with formic acid; mobile phase B = ACN; flow rate = 0.36 mL/min; injection volume = 3 μL; column temperature = 35 °C; autosampler temperature = 22 °C; detection wavelength = 258 nm, and a gradient of *t* = 0 min, 20% B, *t* = 1 min, 20% B, *t* = 16 min, 55% B, *t* = 17 min, 20% B, re-equilibration = 3 min.

### 4.4 Preparation of Sample Solutions

#### 4.4.1 AQbD Study Samples

The solution of cabotegravir was prepared in a mixture of ACN and stress medium 1 M HCl (1:1, v/v) and stored at 50 °C for a few days to generate degradation products **DP1**, **DP2**, and **DP3**. The acidic stress samples were neutralized and diluted before LC analysis to the final concentration of cabotegravir (*c*_cabotegravir_ ∼ 0.5 mg/mL). Degradation product **DP4** was formed after storing cabotegravir in a mixture of ACN and stress medium 0.03% H_2_O_2_ (1:1, v/v) at room temperature for 1 h. In the scouting phase, a mixture of both stress solutions was additionally spiked with related substances **HICBG**, **dFCBG**, **2CBG**, and **4CBG** at concentrations of 0.15% according to cabotegravir concentration (*c*_cabotegravir_ ∼ 0.5 mg/mL) and used for LC analysis. As **DP4** was isolated during AQbD development of the method, the sample used for the screening and optimization phase was prepared by spiking of the acidic stress sample with related substances (**HICBG**, **dFCBG**, **2CBG**, and **4CBG**) and degradation product **DP4**.

#### 4.4.2 Analytical Method Validation Samples

Samples for analytical method validation were prepared in a mixture of ACN and purified water (1:1, v/v). For linearity, a solution of cabotegravir with a concentration of about 0.5 mg/mL was prepared in two replicates and subsequently diluted to achieve concentrations of about 2.5 μg/mL. A solution with concentration about 2.5 μg/mL was further diluted to get solutions with concentrations around 1.0; 0.9; 0.75; 0.6; 0.5; 0.4; and 0.25 μg/mL, respectively.

For the linearity of impurities, a solution of each impurity with a concentration of 20 μg/mL was prepared in two replicates. The solution was further diluted to get solutions with concentrations of approximately 1.0, 0.9, 0.75, 0.6, 0.5, 0.4, and 0.25 μg/mL, respectively. For confirmation of the detection limit, solutions of cabotegravir and all impurities with a concentration 0.125 μg/mL were injected six times. For confirmation of the quantification limit, solutions of cabotegravir and all impurities with a concentration 0.25 μg/mL were injected six times. For accuracy of the method, a spiked sample of cabotegravir with all four related substances as well as all four degradation products with levels of approximately 0.05%, 0.15%, and 0.18% of each impurity in regard to the concentration of cabotegravir in the sample solution were prepared in three replicates (*c*_cabotegravir_ ∼ 0.5 mg/mL).

## Supporting Information Available

Predicted pH curves of cabotegravir made by MarvinSketch; method scouting chromatograms; DoE from method scouting experiments; DoE from method screening experiments; DoE from method optimization experiments; robustness study design; results of robustness stress study; and results for determination of method linearity in analytical method validation for all impurities and cabotegravir (PDF)

## References

[ref1] Han Y.; Mesplède T.; Wainberg M. A. Cabotegravir. HIV Integrase Inhibitor, Anti-HIV Agent. Drugs Future 2015, 40 (11), 705–715. 10.1358/dof.2015.040.11.2383040.

[ref2] Kovač L.; Časar Z. A Literature Review of the Patent Application Publications on Cabotegravir – an HIV Integrase Strand Transfer Inhibitor. Expert Opin. Ther. Pat. 2020, 30 (3), 195–208. 10.1080/13543776.2020.1717470. 31944142

[ref3] GlaxoSmithKline Home Page. https://www.gsk.com/en-gb/media/press-releases/viiv-healthcare-announces-first-global-regulatory-approval-of-cabenuva-the-first-complete-long-acting-regimen-for-the-treatment-of-hiv/ (accessed 2021–12–20).

[ref4] U.S. Food and Drug Administration Home Page. https://www.fda.gov/news-events/press-announcements/fda-approves-first-extended-release-injectable-drug-regimen-adults-living-hiv (accessed 2021–12–20).

[ref5] European Medicines Agency Home Page. https://www.ema.europa.eu/en/news/first-long-acting-injectable-antiretroviral-therapy-hiv-recommended-approval (accessed 2021–12–20).

[ref6] ViiV Healthcare Home Page. https://viivhealthcare.com/hiv-news-and-media/news/press-releases/2021/may/viiv-healthcare-initiates-rolling/ (accessed 2021–12–20).

[ref7] Assessment Report of Vocabria (International non-proprietary name: cabotegravir). European Medicines Agency, 2020. https://www.ema.europa.eu/en/documents/assessment-report/vocabria-epar-public-assessment-report_en.pdf (accessed 2021–12–20).

[ref8] Kovač L.; Črnugelj M.; Roškar R.; Trdan Lušin T.; Časar Z. Understanding of Cabotegravir Degradation Through Isolation and Characterization of Key Degradation Products and Kinetic Studies. J. Pharm. Biomed. Anal. 2021, 201, 114096 10.1016/j.jpba.2021.114096. 33957367

[ref9] Food and Drug Administration, U.S. Department of Health and Human Services. Analytical Procedures and Methods Validation for Drugs and Biologics - Guidance for Industry; U.S. Food and Drug Administration, 2015. https://www.fda.gov/files/drugs/published/Analytical-Procedures-and-Methods-Validation-for-Drugs-and-Biologics.pdf (accessed 2021–12–20).

[ref10] Persich P.; Hellings M.; Jhajra S.; Phalke P.; Vanhoutte K. A Model Approach for Developing Stability-Indicating Analytical Methods, In Methods for Stability Testing of Pharmaceuticals; Bajaj S., Singh S., Eds.; Humana Press, 2018; pp 99–121. 10.1007/978-1-4939-7686-7_5.

[ref11] Vogt F. G.; Kord A. S. Development of Quality-By-Design Analytical Methods. J. Pharm. Sci. 2011, 100 (3), 797–812. 10.1002/jps.22325. 21280050

[ref12] Rozet E.; Lebrun P.; Debrus B.; Boulanger B.; Hubert P. Design Spaces for Analytical Methods. Trends Anal. Chem. 2013, 42, 157–167. 10.1016/j.trac.2012.09.007.

[ref13] Parr M. K.; Schmidt A. H. Life Cycle Management of Analytical Methods. J. Pharm. Biomed. Anal. 2018, 147, 506–517. 10.1016/j.jpba.2017.06.020. 28666555

[ref14] Tome T.; Žigart N.; Časar Z.; Obreza A. Development and Optimization of Liquid Chromatography Analytical Methods by Using AQbD Principles: Overview and Recent Advances. Org. Process Res. Dev. 2019, 23 (9), 1784–1802. 10.1021/acs.oprd.9b00238.

[ref15] International Council for Harmonisation. ICH Guidelines, Quality Guidelines, Q14 Analytical Procedure Development, Q2(R2)/Q14 EWG. International Council for Harmonisation, 2018. https://database.ich.org/sites/default/files/Q2R2-Q14_EWG_Concept_Paper.pdf (accessed 2021–12–20).

[ref16] Tome T.; Časar Z.; Obreza A. Development of a Unified Reversed-Phase HPLC Method for Efficient Determination of EP and USP Process-Related Impurities in Celecoxib Using Analytical Quality by Design Principles. Molecules 2020, 25 (4), 809 10.3390/molecules25040809. PMC7070322 32069880

[ref17] Tome T.; Obreza A.; Časar Z. Developing an Improved UHPLC Method for Efficient Determination of European Pharmacopeia Process-Related Impurities in Ropinirole Hydrochloride Using Analytical Quality by Design Principles. Molecules 2020, 25 (11), 2691 10.3390/molecules25112691. PMC7321168 32531959

[ref18] Žigart N.; Časar Z. Development of a Stability-Indicating Analytical Method for Determination of Venetoclax Using AQbD Principles. ACS Omega 2020, 5 (28), 17726–17742. 10.1021/acsomega.0c02338. 32715260 PMC7377371

[ref19] LoBrutto R.; Patel T. Method Validation. In HPLC for Pharmaceutical Scientists; Kazakevich Y., LoBrutto R., Eds.; John Wiley & Sons, 2007; pp 455–502. 10.1002/9780470087954.ch9.

[ref20] Vejendla A.; Talari S.; Moturu R.; Boddapati S. N. M.; Kola A. E. Method development and validation for Cabotegravir and Rilpivirine by using HPLC and its degradants are characterized by LCMS and FTIR. Futur. J. Pharm. Sci. 2021, 7, 226 10.1186/s43094-021-00355-8.

[ref21] Atkinson A. C; Donev A. N.; Tobias R. D. Optimum Experimental Designs, with SAS; Oxford University Press, 2007.

[ref22] Lundstedt T.; Seifert E.; Abramo L.; Thelin B.; Nyström Å.; Pettersen J.; Bergman R. Experimental Design and Optimization. Chemom. Intell. Lab. Syst. 1998, 42 (1–2), 3–40. 10.1016/S0169-7439(98)00065-3.

[ref23] Maraš N.; Selič L.; Čusak A. Processes for Preparing Dolutegravir and Cabotegravir and Analogues Thereof. US Patent US10335394B2, 2019.

