# Supplementary material for: Development of an Analytical Method for Determination of Related Substances and Degradation Products of Cabotegravir Using Analytical Quality by Design Principles
Source: ACS Omega. 2022 Mar 4;7(10):8896–905. doi: 10.1021/acsomega.1c07260 (PMC8928553; doi:10.1021/acsomega.1c07260)
Supplement: Supplementary file 1 — ao1c07260_si_001.pdf [file ao1c07260_si_001.pdf]

SUPPORTING INFORMATION

Development of an analytical method for determination of related substances and degradation products of cabotegravir using AQbD principles

Lidija Kovač [a,b], Zdenko Časar [a,b], Tina Trdan-Lušin [a,b] and Robert Roškar [b,*]

[a.] *Lek Pharmaceuticals d.d., Sandoz Development Center Slovenia, Analytics Department, SI- 1526 Ljubljana, Slovenia.*
[b.] *University of Ljubljana, Faculty of Pharmacy, Aškerčeva cesta 7, SI-1000, Ljubljana, Slovenia. E-mail: robert.roskar@ffa.uni-lj.si*

LIST OF SUPPORTING INFORMATION

Page	Content
S_3	Figure S_1. Predicted pH curves of cabotegravir made by MarvinSketch
S_4	Figure S_2. Method scouting chromatogram - column HSS T3, 1.8 µm, 150 × 2.1 mm, T_{column}: 30 °C, gradient time 20 minutes, different pH of mobile phase A
S_5	Figure S_3. Method scouting chromatogram - column HSS T3, 1.8 µm, 150 × 2.1 mm, T_{column}: 30 °C, pH of mobile phase A = 2, different gradient time
S_6	Figure S_4. Method scouting chromatogram - column HSS T3, 1.8 µm, 150 × 2.1 mm, gradient time 20 minutes, pH of mobile phase A = 5, different T_{column}
S_7	Figure S_5. Method scouting chromatogram - column XBridge C18, 3.5 µm, 150 × 4.6 mm, gradient time 20 minutes, pH of mobile phase A = 2, T_{column}: 40 °C
S_7	Figure S_6. Method scouting chromatogram - column XBridge C18, 3.5 µm, 150 × 4.6 mm, gradient time 5 minutes, pH of mobile phase A = 2, T_{column}: 30 °C
S_8	Table S_1. DoE from method scouting experiment – part 1
S_9	Table S_2. DoE from method scouting experiment – part 2
S_{10}	Table S_3. DoE from method screening experiment – part 1
S_{11}	Table S_4. DoE from method screening experiment – part 2
S_{12}	Table S_5. DoE from method optimization experiment
S_{13}	Table S_6. Robustness study design
S_{14}	Table S_7. Results of robustness stress study_Run No. 1
S_{14}	Table S_8. Results of robustness stress study_Run No. 2
S_{14}	Table S_9. Results of robustness stress study_Run No. 3
S_{15}	Table S_{10}. Results of robustness stress study_Run No. 4
S_{15}	Table S_{11}. Results of robustness stress study_Run No. 5
S_{15}	Table S_{12}. Results of robustness stress study_Run No. 6
S_{16}	Table S_{13}. Results of robustness stress study_Run No. 7
S_{16}	Table S_{14}. Results of robustness stress study_Run No. 8
S_{16}	Table S_{15}. Results of robustness stress study_Run No. 9
S_{17}	Table S_{16}. Results of robustness stress study_Run No. 10
S_{17}	Table S_{17}. Results of robustness stress study_Run No. 11
S_{17}	Table S_{18}. Results of robustness stress study_Run No. 12
S_{18}	Table S_{19}. Results of robustness stress study_Run No. 13
S_{18}	Table S_{20}. Results of robustness stress study_Run No. 14
S_{18}	Table S_{21}. Results of robustness stress study_Run No. 15
S_{19}	Table S_{22}. Results of robustness stress study_Run No. 16
S_{19}	Table S_{23}. Results of robustness stress study_Run No. 17
S_{19}	Table S_{24}. Results of robustness stress study_Run No. 18
S_{20}	Table S_{25}. Results of robustness stress study_Run No. 19
S_{20}	Table S_{26}. Results of robustness stress study_Run No. 20
S_{21}	Table S_{27} - Linearity results for DP1 from analytical method validation
S_{21}	Table S_{28} - Linearity results for DP2 from analytical method validation
S_{21}	Table S_{29} - Linearity results for DP3 from analytical method validation
S_{22}	Table S_{30} - Linearity results for DP4 from analytical method validation
S_{22}	Table S_{31} - Linearity results for dFCBG from analytical method validation
S_{22}	Table S_{32} - Linearity results for 2CBG from analytical method validation
S_{23}	Table S_{33} - Linearity results for 4CBG from analytical method validation
S_{23}	Table S_{34} - Linearity results for HICBG from analytical method validation
S_{23}	Table S_{35} - Linearity results for cabotegravir from analytical method validation

Figure S₁. Predicted pH curves of *cabotegravir* made by MarvinSketch

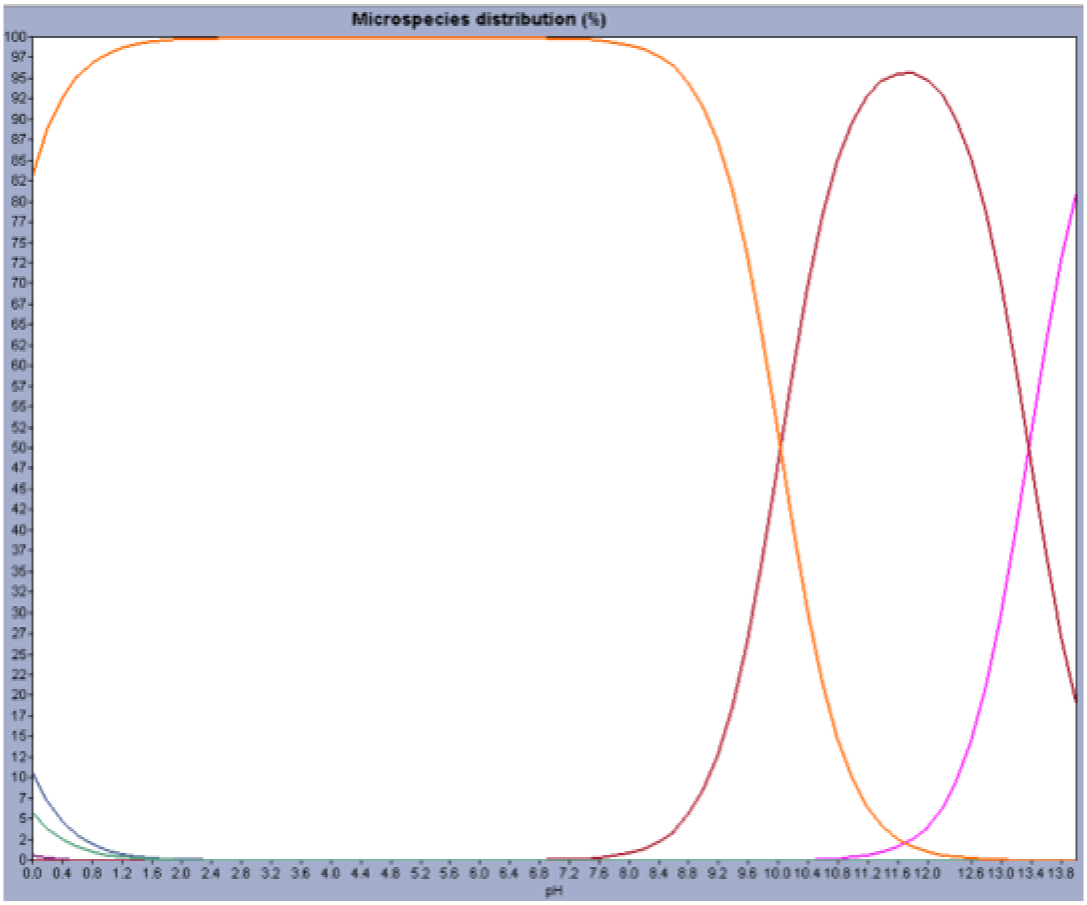

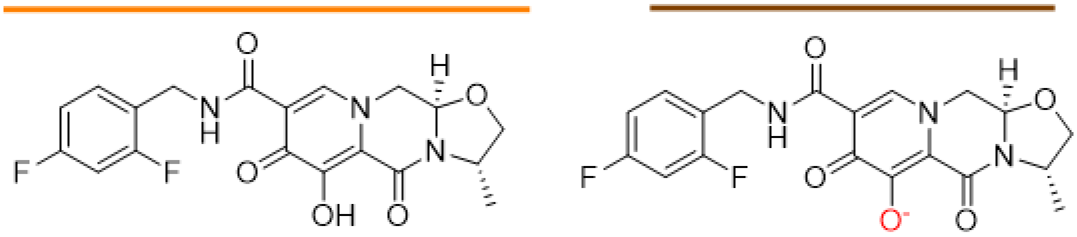

Figure S₂. Method scouting chromatogram - column HSS T3, 1.8 µm, 150 × 2.1 mm, T_{column}: 30 °C, gradient time 20 minutes, different pH of mobile phase A

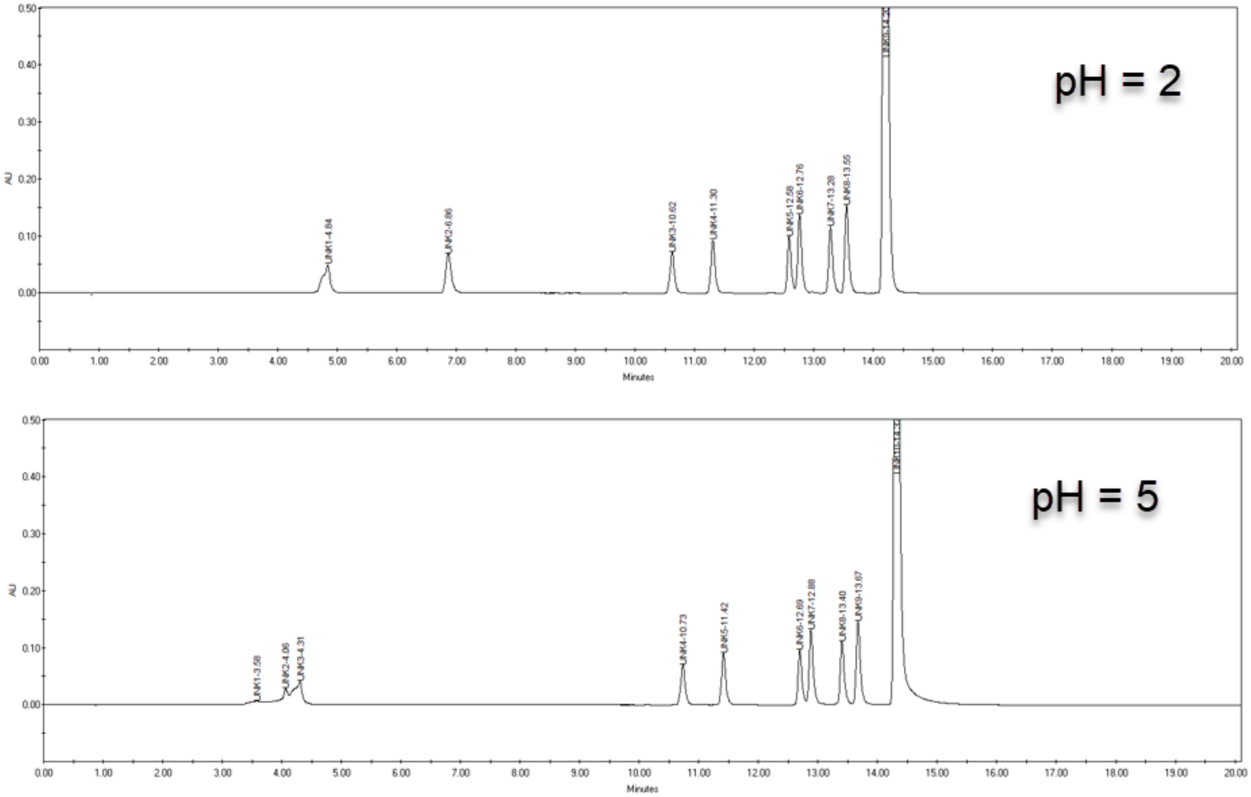

Figure S₃. Method scouting chromatogram – column HSS T3, 1.8 µm, 150 × 2.1 mm, T_{column}: 30 °C, pH of mobile phase A = 2, different gradient time

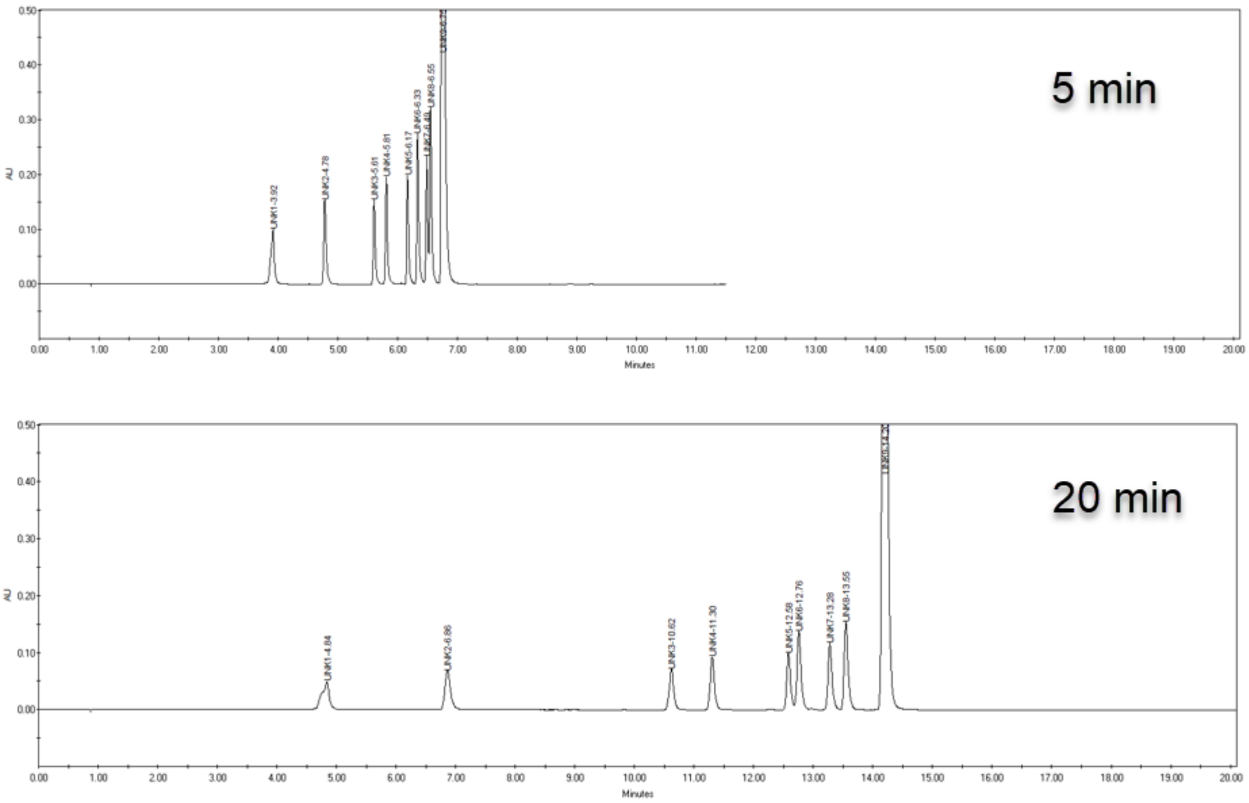

Figure S₄. Method scouting chromatogram - column HSS T3, 1.8 µm, 150 × 2.1 mm, gradient time 20 minutes, pH of mobile phase A = 5, different T_{column}

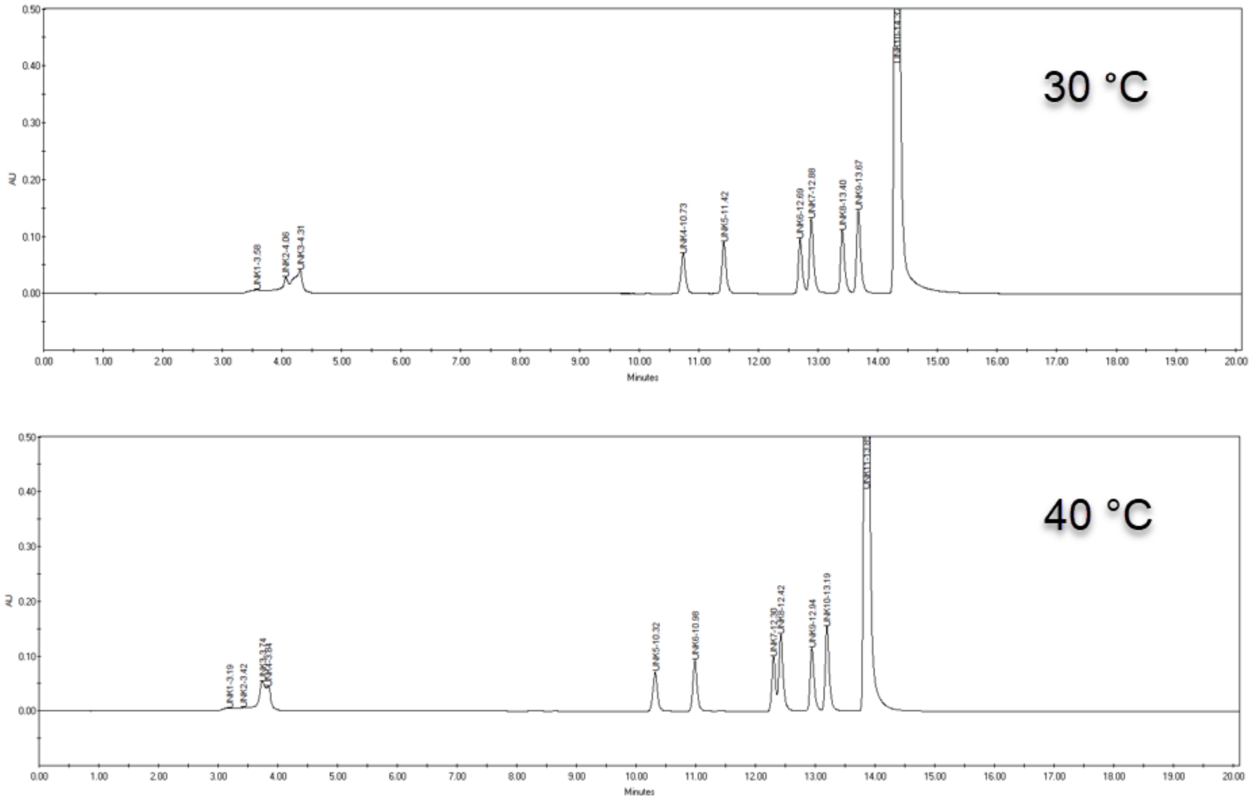

Figure S5. Method scouting chromatogram – column XBridge C18, 3.5 µm, 150 × 4.6 mm, gradient time 20 minutes, pH of mobile phase A = 2, T_{column}: 40 °C

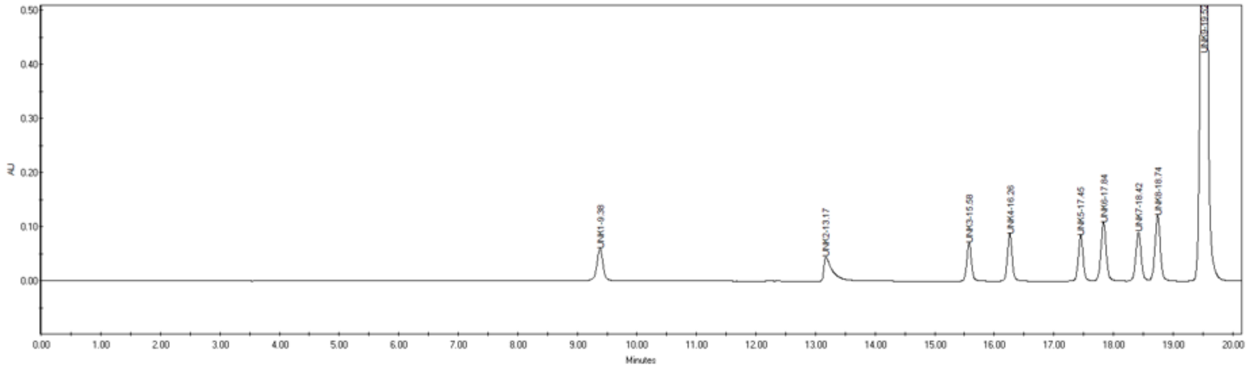

Figure S6. Method scouting chromatogram - column XBridge C18, 3.5 µm, 150 × 4.6 mm, gradient time 5 minutes, pH of mobile phase A = 2, T_{column}: 30 °C

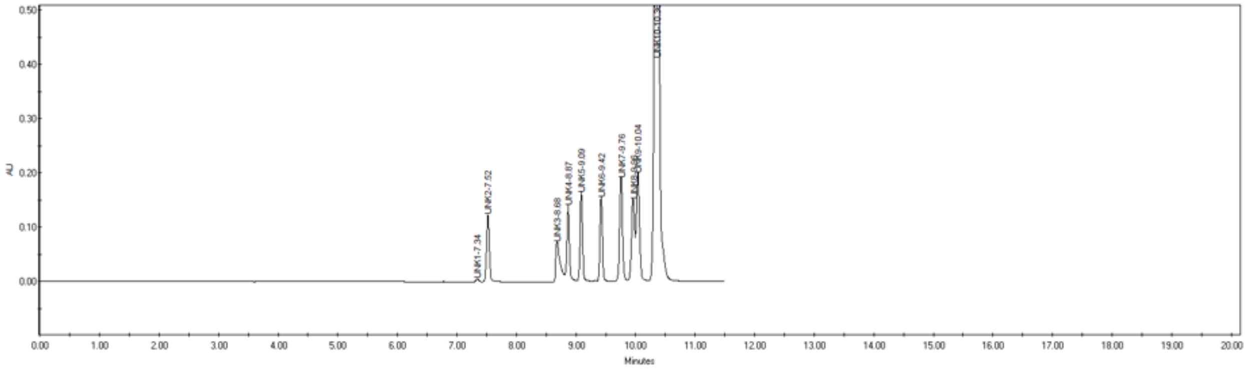

Table S_1. DoE from method scouting experiment – *part 1*

Run No.	Strong Solvent Type (*)	pH (*)	Column Type (*)
Condition Column - 1	MeOH	2.00	XBridge C8
Condition Column - 2	MeOH	2.00	XBridge C18
1	MeOH	2.00	XBridge C8
2	MeOH	2.00	XBridge C18
3	MeOH	2.00	XBridge C18
Condition Column - 3	MeOH	4.00	XBridge C8
Condition Column - 4	MeOH	4.00	XBridge C18
4	MeOH	4.00	XBridge C8
5	MeOH	4.00	XBridge C18
6	MeOH	4.00	XBridge C8
Condition Column - 5	ACN	2.00	XBridge C8
Condition Column - 6	ACN	2.00	XBridge C18
7	ACN	2.00	XBridge C8
8	ACN	2.00	XBridge C18
9	ACN	2.00	XBridge C18
Condition Column - 7	ACN	4.00	XBridge C8
Condition Column - 8	ACN	4.00	XBridge C18
10	ACN	4.00	XBridge C8
11	ACN	4.00	XBridge C18
Condition Column - 9	ACN	4.00	XBridge C8
Condition Column - 10	ACN	4.00	XBridge C18

Table S_2. DoE from method scouting experiment – *part 2*

Run No.	Strong Solvent Type (*)	pH (*)	Column Type (*)
Condition Column - 1	MeOH	2.00	UPLC BEH Phenyl (fenil-heksil)
Condition Column - 2	MeOH	2.00	UPLC HSS T3
1	MeOH	2.00	UPLC BEH Phenyl (fenil-heksil)
2	MeOH	2.00	UPLC HSS T3
3	MeOH	2.00	UPLC HSS T3
Condition Column - 3	MeOH	4.00	UPLC BEH Phenyl (fenil-heksil)
Condition Column - 4	MeOH	4.00	UPLC HSS T3
4	MeOH	4.00	UPLC BEH Phenyl (fenil-heksil)
5	MeOH	4.00	UPLC HSS T3
6	MeOH	4.00	UPLC BEH Phenyl (fenil-heksil)
Condition Column - 5	ACN	2.00	UPLC BEH Phenyl (fenil-heksil)
Condition Column - 6	ACN	2.00	UPLC HSS T3
7	ACN	2.00	UPLC BEH Phenyl (fenil-heksil)
8	ACN	2.00	UPLC HSS T3
9	ACN	2.00	UPLC HSS T3
Condition Column - 7	ACN	4.00	UPLC BEH Phenyl (fenil-heksil)
Condition Column - 8	ACN	4.00	UPLC HSS T3
10	ACN	4.00	UPLC BEH Phenyl (fenil-heksil)
11	ACN	4.00	UPLC HSS T3
Condition Column - 9	ACN	4.00	UPLC BEH Phenyl (fenil-heksil)
Condition Column - 10	ACN	4.00	UPLC HSS T3

Table S$_3$. DoE from method *screening* experiment – *part 1*

Run No.	Pump Flow Rate (mL/min)	Final % Strong Solvent (%)	pH (*)
Condition Column - 1	0.300	60.0	3.50
1	0.200	60.0	3.50
2	0.400	60.0	3.50
3	0.200	90.0	3.50
4	0.200	50.0	3.50
5	0.300	50.0	3.50
6	0.400	90.0	3.50
7	0.400	70.0	3.50
8	0.300	50.0	3.50
Condition Column - 2	0.300	70.0	4.00
9	0.300	70.0	4.00
10	0.400	50.0	4.00
11	0.300	70.0	4.00
12	0.400	50.0	4.00
Condition Column - 3	0.300	60.0	4.50
13	0.400	60.0	4.50
14	0.200	90.0	4.50
15	0.200	50.0	4.50
16	0.400	90.0	4.50
17	0.400	50.0	4.50
Condition Column - 4	0.300	50.0	4.50

Table S_4. DoE from method *screening* experiment – *part 2*

Run No.	Pump Flow Rate (mL/min)	Final % Strong Solvent (%)	pH (*)
Condition Column - 1	0.300	60.0	2.75
1	0.200	60.0	2.75
2	0.400	60.0	2.75
3	0.200	90.0	2.75
4	0.200	50.0	2.75
5	0.300	50.0	2.75
6	0.400	90.0	2.75
7	0.400	70.0	2.75
8	0.300	50.0	2.75
Condition Column - 2	0.300	70.0	3.25
9	0.300	70.0	3.25
10	0.400	50.0	3.25
11	0.300	70.0	3.25
12	0.400	50.0	3.25
Condition Column - 3	0.300	60.0	3.75
13	0.400	60.0	3.75
14	0.200	90.0	3.75
15	0.200	50.0	3.75
16	0.400	90.0	3.75
17	0.400	50.0	3.75
Condition Column - 4	0.300	50.0	3.75

Table S_5. DoE from method optimization experiment

Run No.	Pump Flow Rate (mL/min)	Final % Organic (%)	Oven Temperature (°C)	pH (*)
Condition Column - 1	0.300	90.0	30.0	2.75
1	0.400	90.0	30.0	2.75
2	0.400	50.0	30.0	2.75
3	0.200	90.0	30.0	2.75
4	0.200	50.0	30.0	2.75
5	0.200	50.0	30.0	2.75
6	0.400	50.0	30.0	2.75
7	0.200	90.0	30.0	2.75
Condition Column - 2	0.300	70.0	30.0	3.25
8	0.300	70.0	30.0	3.25
Condition Column - 3	0.300	50.0	30.0	3.75
9	0.200	50.0	30.0	3.75
10	0.400	50.0	30.0	3.75
11	0.200	90.0	30.0	3.75
12	0.400	90.0	30.0	3.75
Condition Column - 4	0.300	70.0	40.0	2.75
13	0.300	70.0	40.0	2.75
Condition Column - 5	0.300	70.0	40.0	3.25
14	0.200	70.0	40.0	3.25
15	0.400	70.0	40.0	3.25
16	0.300	50.0	40.0	3.25
17	0.300	90.0	40.0	3.25
18	0.300	70.0	40.0	3.25
19	0.300	70.0	40.0	3.25
20	0.300	70.0	40.0	3.25
Condition Column - 6	0.300	70.0	40.0	3.75
21	0.300	70.0	40.0	3.75
Condition Column - 7	0.300	50.0	50.0	2.75
22	0.200	50.0	50.0	2.75
23	0.400	50.0	50.0	2.75
24	0.200	90.0	50.0	2.75
25	0.400	90.0	50.0	2.75
Condition Column - 8	0.300	70.0	50.0	3.25
26	0.300	70.0	50.0	3.25
Condition Column - 9	0.300	50.0	50.0	3.75
27	0.200	50.0	50.0	3.75
28	0.400	50.0	50.0	3.75
29	0.200	90.0	50.0	3.75
30	0.400	90.0	50.0	3.75
Condition Column - 10	0.300	90.0	50.0	3.75

Table S_6. Robustness study design

Run No.	Pump Flow rate (ml/min)	Final % organic (%)	Oven temperature (° C)	pH
1	0.320	50.0	30.0	3.25
2	0.320	60.0	30.0	3.25
3	0.400	50.0	30.0	3.25
4	0.400	60.0	30.0	3.25
5	0.360	55.0	30.0	3.25
6	0.320	50.0	30.0	3.75
7	0.320	60.0	30.0	3.75
8	0.400	50.0	30.0	3.75
9	0.400	60.0	30.0	3.75
10	0.360	55.0	30.0	3.75
11	0.320	50.0	40.0	3.25
12	0.320	60.0	40.0	3.25
13	0.400	50.0	40.0	3.25
14	0.400	60.0	40.0	3.25
15	0.360	55.0	40.0	3.25
16	0.320	50.0	40.0	3.75
17	0.320	60.0	40.0	3.75
18	0.400	50.0	40.0	3.75
19	0.400	60.0	40.0	3.75
20	0.360	55.0	40.0	3.75

Table S_7. Results of robustness stress study_Run No. 1

Study Variable Name	Prediction Point Level Setting	
Pump Flow Rate	0.320	
Final % Organic	50.0	
Oven Temperature	30.0	
pH	3.25	
**Response Variable Name**	**Predicted Result**	**Actual result**
$R_{DP4, HICBG}$ - USPResolution	4.4	4.3
$R_{2CBG, 4CBG}$ - USPResolution	3.6	3.7
$R_{4CBG, cabotegravir}$ - USPResolution	3.5	3.3

Table S_8. Results of robustness stress study_Run No. 2

Study Variable Name	Prediction Point Level Setting	
Pump Flow Rate	0.320	
Final % Organic	60.0	
Oven Temperature	30.0	
pH	3.25	
**Response Variable Name**	**Predicted Result**	**Actual result**
$R_{DP4, HICBG}$ - USPResolution	4.4	4.3
$R_{2CBG, 4CBG}$ - USPResolution	3.3	3.3
$R_{4CBG, cabotegravir}$ - USPResolution	2.9	2.9

Table S_9. Results of robustness stress study_Run No. 3

Study Variable Name	Prediction Point Level Setting	
Pump Flow Rate	0.400	
Final % Organic	50.0	
Oven Temperature	30.0	
pH	3.25	
**Response Variable Name**	**Predicted Result**	**Actual result**
$R_{DP4, HICBG}$ - USPResolution	4.3	4.3
$R_{2CBG, 4CBG}$ - USPResolution	3.7	3.7
$R_{4CBG, cabotegravir}$ - USPResolution	3.3	3.3

Table S_{10}. Results of robustness stress study_Run No. 4

Study Variable Name	Prediction Point Level Setting	
Pump Flow Rate	0.400	
Final % Organic	60.0	
Oven Temperature	30.0	
pH	3.25	
**Response Variable Name**	**Predicted Result**	**Actual result**
$R_{DP4, HICBG}$ - USPResolution	4.3	4.3
$R_{2CBG, 4CBG}$ - USPResolution	3.3	3.4
$R_{4CBG, cabotegravir}$ - USPResolution	2.9	3.0

Table S_{11}. Results of robustness stress study_Run No. 5

Study Variable Name	Prediction Point Level Setting	
Pump Flow Rate	0.360	
Final % Organic	55.0	
Oven Temperature	30.0	
pH	3.25	
**Response Variable Name**	**Predicted Result**	**Actual result**
$R_{DP4, HICBG}$ - USPResolution	4.3	4.3
$R_{2CBG, 4CBG}$ - USPResolution	3.5	3.6
$R_{4CBG, cabotegravir}$ - USPResolution	3.2	3.1

Table S_{12}. Results of robustness stress study_Run No. 6

Study Variable Name	Prediction Point Level Setting	
Pump Flow Rate	0.320	
Final % Organic	50.0	
Oven Temperature	30.0	
pH	3.75	
**Response Variable Name**	**Predicted Result**	**Actual result**
$R_{DP4, HICBG}$ - USPResolution	5.5	5.5
$R_{2CBG, 4CBG}$ - USPResolution	3.6	3.7
$R_{4CBG, cabotegravir}$ - USPResolution	3.5	3.2

Table S_{13}. Results of robustness stress study_Run No. 7

Study Variable Name	Prediction Point Level Setting		
Pump Flow Rate	0.320		
Final % Organic	60.0		
Oven Temperature	30.0		
pH	3.75		
**Response Variable Name**		**Predicted Result**	**Actual result**
$R_{DP4, HICBG}$ - USPResolution		5.6	5.5
$R_{2CBG, 4CBG}$ - USPResolution		3.2	3.3
$R_{4CBG, cabotegravir}$ - USPResolution		2.8	2.8

Table S_{14}. Results of robustness stress study_Run No. 8

Study Variable Name	Prediction Point Level Setting		
Pump Flow Rate	0.400		
Final % Organic	50.0		
Oven Temperature	30.0		
pH	3.75		
**Response Variable Name**		**Predicted Result**	**Actual result**
$R_{DP4, HICBG}$ - USPResolution		5.5	5.4
$R_{2CBG, 4CBG}$ - USPResolution		3.6	3.7
$R_{4CBG, cabotegravir}$ - USPResolution		3.2	3.3

Table S_{15}. Results of robustness stress study_Run No. 9

Study Variable Name	Prediction Point Level Setting		
Pump Flow Rate	0.400		
Final % Organic	60.0		
Oven Temperature	30.0		
pH	3.75		
**Response Variable Name**		**Predicted Result**	**Actual result**
$R_{DP4, HICBG}$ - USPResolution		5.5	5.4
$R_{2CBG, 4CBG}$ - USPResolution		3.3	3.4
$R_{4CBG, cabotegravir}$ - USPResolution		2.8	3.0

Table S_{16}. Results of robustness stress study_Run No. 10

Study Variable Name	Prediction Point Level Setting	
Pump Flow Rate	0.360	
Final % Organic	55.0	
Oven Temperature	30.0	
pH	3.75	
**Response Variable Name**	**Predicted Result**	**Actual result**
$R_{DP4,\ HICBG}$ - USPResolution	5.5	5.5
$R_{2CBG,\ 4CBG}$ - USPResolution	3.5	3.5
$R_{4CBG,\ cabotegravir}$ - USPResolution	3.1	3.1

Table S_{17}. Results of robustness stress study_Run No. 11

Study Variable Name	Prediction Point Level Setting	
Pump Flow Rate	0.320	
Final % Organic	50.0	
Oven Temperature	40.0	
pH	3.25	
**Response Variable Name**	**Predicted Result**	**Actual result**
$R_{DP4,\ HICBG}$ - USPResolution	3.9	3.9
$R_{2CBG,\ 4CBG}$ - USPResolution	3.6	3.6
$R_{4CBG,\ cabotegravir}$ - USPResolution	3.5	3.1

Table S_{18}. Results of robustness stress study_Run No. 12

Study Variable Name	Prediction Point Level Setting	
Pump Flow Rate	0.320	
Final % Organic	60.0	
Oven Temperature	40.0	
pH	3.25	
**Response Variable Name**	**Predicted Result**	**Actual result**
$R_{DP4,\ HICBG}$ - USPResolution	3.9	3.9
$R_{2CBG,\ 4CBG}$ - USPResolution	3.3	3.3
$R_{4CBG,\ cabotegravir}$ - USPResolution	2.9	2.8

Table S_{19}. Results of robustness stress study_Run No. 13

Study Variable Name	Prediction Point Level Setting	
Pump Flow Rate	0.400	
Final % Organic	50.0	
Oven Temperature	40.0	
pH	3.25	
**Response Variable Name**	**Predicted Result**	**Actual result**
$R_{DP4, HICBG}$ - USPResolution	3.9	3.9
$R_{2CBG, 4CBG}$ - USPResolution	3.6	3.6
$R_{4CBG, cabotegravir}$ - USPResolution	3.2	3.1

Table S_{20}. Results of robustness stress study_Run No. 14

Study Variable Name	Prediction Point Level Setting	
Pump Flow Rate	0.400	
Final % Organic	60.0	
Oven Temperature	40.0	
pH	3.25	
**Response Variable Name**	**Predicted Result**	**Actual result**
$R_{DP4, HICBG}$ - USPResolution	3.9	3.9
$R_{2CBG, 4CBG}$ - USPResolution	3.3	3.4
$R_{4CBG, cabotegravir}$ - USPResolution	2.8	2.8

Table S_{21}. Results of robustness stress study_Run No. 15

Study Variable Name	Prediction Point Level Setting	
Pump Flow Rate	0.360	
Final % Organic	55.0	
Oven Temperature	40.0	
pH	3.25	
**Response Variable Name**	**Predicted Result**	**Actual result**
$R_{DP4, HICBG}$ - USPResolution	3.9	3.9
$R_{2CBG, 4CBG}$ - USPResolution	3.5	3.5
$R_{4CBG, cabotegravir}$ - USPResolution	3.2	3.0

Table S_{22}. Results of robustness stress study_Run No. 16

Study Variable Name	Prediction Point Level Setting	
Pump Flow Rate	0.320	
Final % Organic	50.0	
Oven Temperature	40.0	
pH	3.75	
**Response Variable Name**	**Predicted Result**	**Actual result**
$R_{DP4, HICBG}$ - USPResolution	4.8	4.8
$R_{2CBG, 4CBG}$ - USPResolution	3.6	3.6
$R_{4CBG, cabotegravir}$ - USPResolution	3.4	3.0

Table S_{23}. Results of robustness stress study_Run No. 17

Study Variable Name	Prediction Point Level Setting	
Pump Flow Rate	0.320	
Final % Organic	60.0	
Oven Temperature	40.0	
pH	3.75	
**Response Variable Name**	**Predicted Result**	**Actual result**
$R_{DP4, HICBG}$ - USPResolution	4.9	4.8
$R_{2CBG, 4CBG}$ - USPResolution	3.2	3.2
$R_{4CBG, cabotegravir}$ - USPResolution	2.8	2.7

Table S_{24}. Results of robustness stress study_Run No. 18

Study Variable Name	Prediction Point Level Setting	
Pump Flow Rate	0.400	
Final % Organic	50.0	
Oven Temperature	40.0	
pH	3.75	
**Response Variable Name**	**Predicted Result**	**Actual result**
$R_{DP4, HICBG}$ - USPResolution	4.9	4.9
$R_{2CBG, 4CBG}$ - USPResolution	3.6	3.6
$R_{4CBG, cabotegravir}$ - USPResolution	3.1	3.1

Table S_{25}. Results of robustness stress study_Run No. 19

Study Variable Name	Prediction Point Level Setting		
Pump Flow Rate	0.400		
Final % Organic	60.0		
Oven Temperature	40.0		
pH	3.75		
**Response Variable Name**		**Predicted Result**	**Actual result**
$R_{DP4, HICBG}$ - USPResolution		4.9	4.9
$R_{2CBG, 4CBG}$ - USPResolution		3.2	3.3
$R_{4CBG, cabotegravir}$ - USPResolution		2.7	2.8

Table S_{26}. Results of robustness stress study_Run No. 20

Study Variable Name	Prediction Point Level Setting		
Pump Flow Rate	0.360		
Final % Organic	55.0		
Oven Temperature	40.0		
pH	3.75		
**Response Variable Name**		**Predicted Result**	**Actual result**
$R_{DP4, HICBG}$ - USPResolution		4.9	4.8
$R_{2CBG, 4CBG}$ - USPResolution		3.4	3.4
$R_{4CBG, cabotegravir}$ - USPResolution		3.1	2.9

Table S_{27}. Linearity results for DP1 from analytical method validation

Target concentration (%)	Concentration (µg/mL; x)	Average peak area (area; y)
0.05	0.259	7258
0.08	0.414	11377
0.10	0.518	14261
0.12	0.621	17209
0.15	0.776	21933
0.18	0.932	26303
0.20	1.035	29747
Correlation coefficient	1.000	
Regression line	y = 28984x – 558	

Table S_{28}. Linearity results for DP2 from analytical method validation

Target concentration (%)	Concentration (µg/mL; x)	Average peak area (area; y)
0.05	0.262	7137
0.08	0.420	11560
0.1	0.524	14543
0.12	0.629	17694
0.15	0.787	22029
0.18	0.944	26179
0.2	1.049	29018
Correlation coefficient	1.000	
Regression line	y = 27865x – 63	

Table S_{29}. Linearity results for DP3 from analytical method validation

Target concentration (%)	Concentration (µg/mL; x)	Average peak area (area; y)
0.05	0.251	5944
0.08	0.402	9688
0.1	0.503	12084
0.12	0.603	14652
0.15	0.754	18358
0.18	0.905	22059
0.2	1.006	24652
Correlation coefficient	1.000	
Regression line	y = 24753x – 300	

Table S_{30}. Linearity results for DP4 from analytical method validation

Target concentration (%)	Concentration (µg/mL; x)	Average peak area (area; y)
0.05	0.268	2253
0.08	0.429	3437
0.1	0.537	4349
0.12	0.644	5257
0.15	0.805	6670
0.18	0.966	8039
0.2	1.074	8808
Correlation coefficient	1.000	
Regression line	y = 8303x - 58	

Table S_{31}. Linearity results for dFCBG from analytical method validation

Target concentration (%)	Concentration (µg/mL; x)	Average peak area (area; y)
0.05	0.259	6648
0.08	0.414	10842
0.1	0.518	13674
0.12	0.621	16296
0.15	0.776	20313
0.18	0.932	24875
0.2	1.035	27947
Correlation coefficient	1.000	
Regression line	y = 27223x – 487	

Table S_{32}. Linearity results for 2CBG from analytical method validation

Target concentration (%)	Concentration (µg/mL; x)	Average peak area (area; y)
0.05	0.262	5871
0.08	0.420	9889
0.1	0.525	12221
0.12	0.629	15090
0.15	0.787	19078
0.18	0.944	22900
0.2	1.049	25575
Correlation coefficient	1.000	
Regression line	y = 25057x - 719	

Table S_{33}. Linearity results for 4CBG from analytical method validation

Target concentration (%)	Concentration (µg/mL; x)	Average peak area (area; y)
0.05	0.269	6159
0.08	0.430	10243
0.1	0.538	13001
0.12	0.645	15631
0.15	0.807	19522
0.18	0.968	24072
0.2	1.076	27019
Correlation coefficient	1.000	
Regression line	y = 25708x – 863	

Table S_{34}. Linearity results for HICBG from analytical method validation

Target concentration (%)	Concentration (µg/mL; x)	Average peak area (area; y)
0.05	0.269	3732
0.08	0.431	5802
0.1	0.538	7537
0.12	0.646	9090
0.15	0.807	11700
0.18	0.969	15085
0.2	1.076	16571
Correlation coefficient	0.998	
Regression line	y = 16291x – 1090	

Table S_{35}. Linearity results for cabotegravir from analytical method validation

Target concentration (%)	Concentration (µg/mL; x)	Average peak area (area; y)
0.05	0.251	5169
0.08	0.402	8800
0.1	0.503	11051
0.12	0.603	13628
0.15	0.754	17145
0.18	0.905	21413
0.2	1.006	23954
Correlation coefficient	0.999	
Regression line	y = 24927x - 1307	
